# Genetic and immunohistochemical profiling of small cell and large cell neuroendocrine carcinomas of the breast

**DOI:** 10.1038/s41379-022-01090-y

**Published:** 2022-05-19

**Authors:** Gregory R. Bean, Saleh Najjar, Sandra J. Shin, Elizabeth M. Hosfield, Jennifer L. Caswell-Jin, Anatoly Urisman, Kirk D. Jones, Yunn-Yi Chen, Gregor Krings

**Affiliations:** 1 Department of Pathology, Stanford University School of Medicine, Stanford, CA USA; 2 Department of Pathology and Laboratory Medicine, Albany Medical College, Albany, NY USA; 3 Department of Pathology, Kaiser Permanente San Francisco Medical Center, San Francisco, CA USA; 4 Department of Medicine, Division of Oncology, Stanford University School of Medicine, Stanford, CA USA; 5 Department of Pathology, University of California San Francisco, San Francisco, CA USA

**Keywords:** Pathology, Breast cancer, Translational research, Cancer genomics, Oncogenes

## Abstract

Neuroendocrine carcinomas (NEC) of the breast are exceedingly rare tumors, which are classified in the WHO system as small cell (SCNEC) and large cell (LCNEC) carcinoma based on indistinguishable features from their lung counterparts. In contrast to lung and enteropancreatic NEC, the genomics of breast NEC have not been well-characterized. In this study, we examined the clinicopathologic, immunohistochemical, and genetic features of 13 breast NEC (7 SCNEC, 4 LCNEC, 2 NEC with ambiguous small versus large cell morphology [ANEC]). Co-alterations of *TP53* and *RB1* were identified in 86% (6/7) SCNEC, 100% (2/2) ANEC, and 50% (2/4) LCNEC. The one SCNEC without *TP53*/*RB1* alteration had other p53 pathway aberrations (*MDM2* and *MDM4* amplification) and was immunohistochemically RB negative. *PIK3CA*/*PTEN* pathway alterations and *ZNF703* amplifications were each identified in 46% (6/13) NEC. Two tumors (1 SCNEC, 1 LCNEC) were *CDH1* mutated. By immunohistochemistry, 100% SCNEC (6/6) and ANEC (2/2) and 50% (2/4) LCNEC (83% NEC) showed RB loss, compared to 0% (0/8) grade 3 neuroendocrine tumors (NET) (*p* < 0.001) and 38% (36/95) grade 3 invasive ductal carcinomas of no special type (IDC-NST) (*p* = 0.004). NEC were also more often p53 aberrant (60% vs 0%, *p* = 0.013), ER negative (69% vs 0%, *p* = 0.005), and GATA3 negative (67% vs 0%, *p* = 0.013) than grade 3 NET. Two mixed NEC had IDC-NST components, and 69% (9/13) of tumors were associated with carcinoma in situ (6 neuroendocrine DCIS, 2 non-neuroendocrine DCIS, 1 non-neuroendocrine LCIS). NEC and IDC-NST components of mixed tumors were clonally related and immunophenotypically distinct, lacking ER and GATA3 expression in NEC relative to IDC-NST, with RB loss only in NEC of one ANEC. The findings provide insight into the pathogenesis of breast NEC, underscore their classification as a distinct tumor type, and highlight genetic similarities to extramammary NEC, including highly prevalent p53/RB pathway aberrations in SCNEC.

## Introduction

Neuroendocrine carcinomas (NEC) of the breast are rare high-grade malignancies that are poorly understood biologically and clinically. Although neuroendocrine differentiation of breast tumors has long been recognized, classification has been problematic and has continued to shift over the years, with the most recent World Health Organization (WHO) classification (5^th^ edition) based on a consensus that terminology should be more uniform across anatomic sites^1–4^. Accordingly, the WHO has defined NEC of the breast as small cell neuroendocrine carcinoma (SCNEC) and large cell neuroendocrine carcinoma (LCNEC), emphasizing that these tumors are histologically and immunohistochemically indistinguishable from their respective lung counterparts^2,5^. SCNEC has been recognized as a distinct and clinically aggressive breast cancer subtype for many years, although most published literature is based on case reports and small series^6–9^. The classification of LCNEC as a separate entity in the breast is again acknowledged in the most recent WHO edition, yet debated^10^, and clinical and pathologic features of these rare tumors remain largely uncharacterized.

NEC of the breast should be distinguished from neuroendocrine tumors (NET), which are morphologically distinct from NEC and are usually Nottingham grade 1 or 2^5,11^. However, it is recognized that some Nottingham grade 3 neuroendocrine neoplasms (NEN) of the breast do not resemble SCNEC or LCNEC morphologically^1^. These tumors lack high-grade nuclei, necrosis, and other nucleocytologic features of SCNEC or LCNEC despite areas of high mitotic activity and diffuse neuroendocrine marker expression. It is unclear if such tumors should be classified as NEC or grade 3 NET in the current WHO classification. In the enteropancreatic system, it has been well-established that high-grade (G3) NET can be challenging to differentiate from NEC histopathologically, yet these neoplasms are biologically and clinically distinct from one another^12–15^. Whether a similar paradigm exists for NEC and these other grade 3 NEN in the breast is unknown.

In this study, we comprehensively characterized a cohort of breast NEC (SCNEC, LCNEC, and NEC with features ambiguous between small and large cell morphology) by capture-based next-generation sequencing (NGS) and immunohistochemistry in order to identify molecular drivers and to determine if these rare aggressive tumors share pathogenetic features with NEC arising in other sites. NEC and invasive ductal carcinoma of no special type (IDC-NST) components of mixed tumors were separately analyzed to assess shared clonality and immunophenotypic divergence between the components. The immunoprofiles of NEC were additionally compared to other grade 3 NEN that morphologically were not considered to meet NEC criteria (including high-grade NET), and to grade 3 IDC-NST. Our results provide novel insights into the pathogenesis of breast SCNEC and LCNEC and highlight genetic similarities to extramammary NEC, including highly prevalent p53/RB pathway aberrations in SCNEC. Overall the findings support the separate classification of breast NEC, especially SCNEC, and suggest that LCNEC may be a more heterogeneous group.

## Materials and methods

### Study population and tumor classification

With institutional review board approval, the pathology archives of University of California San Francisco (UCSF), Stanford University, and Kaiser Permanente (San Francisco, CA) were searched for cases of Nottingham grade 3 NEN of the breast. This was supplemented by the consultation service of one of the authors (S.J.S.); three SCNEC were described in part in a prior report^6^. NEC (*n* = 11) and NEC components of mixed NEC/IDC-NST tumors (*n* = 2) comprising the study population were classified as SCNEC or LCNEC based on independent review by two pulmonary pathologists experienced in the diagnosis of neuroendocrine carcinoma (A.U. and K.D.J.), who classified them based on morphologic criteria used in the lung. Two tumors with discordant classification as SCNEC or LCNEC were classified as NEC with features ambiguous for small cell versus large cell morphology (ANEC1 and ANEC2). All NEC expressed at least one neuroendocrine marker, and diffuse (>90%) staining with synaptophysin and/or chromogranin was required for LCNEC. Of these 13 tumors, eleven (6 SCNEC, 2 ANEC, and 3 LCNEC) were analyzed by DNA sequencing using the UCSF500 assay, and two (1 SCNEC, 1 LCNEC) were submitted for FoundationOne tumor-only sequencing for clinical purposes (Foundation Medicine, Cambridge, MA).

Eight Nottingham grade 3 NEN with diffuse neuroendocrine morphology and extensive (>90%) synaptophysin and/or chromogranin expression that did not show characteristic cytomorphologic features of either SCNEC or LCNEC of the lung were identified. We opted to classify these tumors as grade 3 NET for purposes of comparison to NEC (see *Results* for additional histologic description of these tumors). Nottingham grade 3 invasive carcinomas with less than diffuse neuroendocrine differentiation, including <90% synaptophysin and/or chromogranin expression, were classified as invasive ductal carcinomas with neuroendocrine differentiation (IDC-NED) (*n* = 2) or invasive lobular carcinoma with neuroendocrine differentiation, solid pattern (ILC-NED) (*n* = 1)^16^.

Clinical information was obtained from electronic medical records when available. All tumors were confirmed to be mammary in origin based on clinical history, imaging, and pathologic findings.

### Capture-based next generation DNA sequencing

Matched tumor and normal tissue were selected from nine pure NEC (6 SCNEC, 1 ANEC, 2 LCNEC) and two mixed NEC/IDC-NST (ANEC2 and LCNEC1) for capture-based NGS (*n* = 11). For the two mixed NEC/IDC-NST tumors, DNA from NEC and IDC-NST areas was macrodissected and analyzed separately. For patients treated with chemotherapy, only pre-treatment tumor was tested by NGS. Sequencing libraries were prepared from genomic DNA extracted from punch biopsies or macrodissected unstained sections from formalin-fixed paraffin-embedded tissue. Target enrichment was performed by hybrid capture using a custom oligonucleotide library. Capture-based NGS was performed at the UCSF Clinical Cancer Genomics Laboratory, using an assay (UCSF500 panel) that targets the coding regions of 480 cancer-related genes, select introns from ~40 genes, and the *TERT* promoter with a total sequencing footprint of 2.8 Mb (Supplementary Table S1). Sequencing was performed on a HiSeq 2500 (Illumina, San Diego, CA). Duplicate sequencing reads were removed computationally to allow for accurate allele frequency determination and copy number calling. The analysis was based on the human reference sequence UCSC build hg19 (NCBI build 37), using the following software packages: BWA: 0.7.10-r789, Samtools: 1.1 (using htslib 1.1), Picard tools: 1.97 (1504), GATK: 2014.4-3.3.0-0-ga3711, CNVkit: 0.3.3, Pindel: 0.2.5a7, SATK: 2013.1-10- gd6fa6c3, Annovar: v2015Mar22, Freebayes: 0.9.20 and Delly: 0.5.9^17–25^. Only insertions/deletions (indels) up to 100 bp in length were included in the mutational analysis. Somatic single nucleotide variants and indels were visualized and verified using Integrated Genome Viewer (Broad Institute, Cambridge, MA, USA). Tumor mutational burden was quantified, reflecting somatic synonymous and nonsynonymous single nucleotide variants and small indels in coding regions and splice sites. Genome-wide copy number analysis based on on-target and off-target reads was performed by CNVkit and Nexus Copy Number (Biodiscovery, Hawthorne, CA, USA).

### Immunohistochemistry and in situ hybridization

The following antibodies were used for immunohistochemistry: synaptophysin (polyclonal, 1:100, Cell Marque, Rocklin, CA, USA), chromogranin (LK2H10, 1:4, Cell Marque), INSM1 (A-8, 1:200, Santa Cruz Biotechnology, Santa Cruz, CA, USA), NSE (22C9, undiluted, Leica Biosystems), TTF1 (8G7G3/1, 1:500, DAKO, Santa Clara, CA, USA), ER (SP1, undiluted, Ventana, Tucson, AZ, USA), PR (1E2, undiluted, Ventana), HER2 (4B5, undiluted, Ventana), Ki-67 (MIB1, 1:50, DAKO), E-cadherin (HECD-1, 1:100, Invitrogen, Carlsbad, CA, USA), GATA3 (L50-823, 1:50, Biocare Medical, Concord, CA, USA), RB (G3-245, 1:100, BD Biosciences, Franklin Lakes, NJ, USA), p53 (DO7, undiluted, Leica Biosystems), ATRX (polyclonal, 1:100, Sigma, St. Louis, MO, USA), and p16 (E6H4, 1:2, Ventana). RNA in situ hybridization for human papillomavirus (HPV) was HR18 (848568, ACD Bio, Newark, CA, USA). Antigen retrieval was as follows: for synaptophysin, INSM1, NSE, Ki-67, GATA3, Rb, p53, and p16, BOND ER2 (Leica Biosystems); for chromogranin, TTF1, E-cadherin, and ATRX, BOND ER1 (Leica Biosystems); for ER, PR, and HER2, Ventana CC1 (Ventana); and for HPV, RNAprotease.

For ER, PR, and HER2, positive staining was defined according to ASCO/CAP guidelines^26,27^. HER2 FISH testing was performed using the Abbott Vysis Pathvysion (Des Plaines, IL, USA) FDA-cleared kit, performed per manufacturer recommendations, and scored and interpreted according to ASCO/CAP guidelines^27^. For synaptophysin, chromogranin, INSM1, NSE, GATA3, and TTF1, positive staining was segregated as ≥90%, 50–89%, and 1–49%. For p16, diffuse (≥90%) strong nuclear and cytoplasmic staining (i.e., “block-positive”) was considered overexpressed, patchy weak to strong cytoplasmic staining was considered normal/wild-type pattern, and lack of staining was considered negative^28^. For RB, patchy to diffuse nuclear staining was considered intact, and lack of nuclear staining was considered negative. For p53, diffuse (≥90%) moderate to strong nuclear staining was considered positive/aberrant, no nuclear staining was considered negative/null, and patchy heterogeneous nuclear staining was considered wild-type pattern.

### Statistical analysis

Statistical analysis was performed using Fisher exact test, using a significance level of *p* < 0.05. The degree of interobserver agreement was quantified by kappa.

## Results

### Diagnostic histologic and immunophenotypic features and classification of neuroendocrine carcinomas

**Fig. 1:**
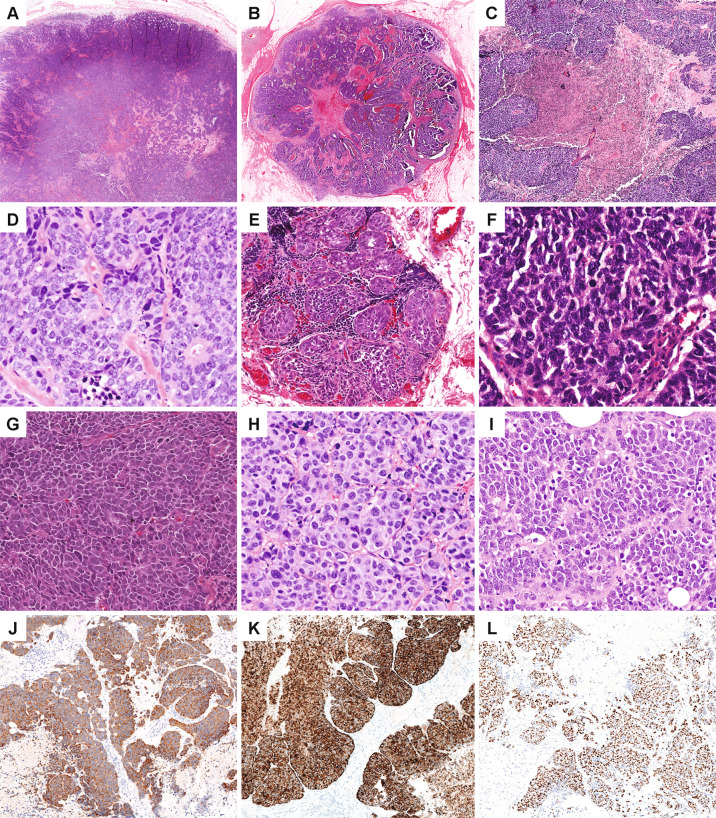
Breast neuroendocrine carcinomas. Neuroendocrine carcinomas exhibited organoid, nested, trabecular, and/or sheet-like growth patterns; some tumors demonstrated circumscribed or expansile borders (**A**, LCNEC1; **B**, SCNEC1). **C** Geographic tumor necrosis was often present (SCNEC3). **D** Single-cell necrosis and apoptotic bodies were prevalent (SCNEC7). **E** The majority of cases showed focal in situ carcinoma with neuroendocrine features similar to the invasive tumor (SCNEC4). **F** Classic cytologic features were used to diagnose small cell neuroendocrine carcinoma, including high nuclear:cytoplasmic ratio, indistinct nucleoli, and nuclear molding (SCNEC4). **G** Rare cases demonstrated more spindled cytomorphology (SCNEC1). **H** Features supportive of large cell neuroendocrine carcinoma included abundant cytoplasm, distinct and more frequent nucleoli, and well-defined cellular borders (LCNEC2). **I** Occasional cases demonstrated scant cytoplasm yet prominent nucleoli; such NEC with mixed features were designated as ambiguous between small cell and large cell neuroendocrine carcinoma (ANEC1). Cases were positive for neuroendocrine markers by immunohistochemistry, including synaptophysin (**J**), chromogranin (**K**), and INSM1 (**L**) (LCNEC2).

All breast NEC and NEC components of mixed NEC/IDC-NST showed morphologic features typical of poorly-differentiated NEC in the lung and were Nottingham grade 3, with high nuclear grade, high mitotic index, and poor glandular differentiation (Fig. 1 and Table 1). Individual cytomorphologic features including cell size, nuclear, nucleolar, and cytoplasmic features, tumor growth pattern, and the presence of geographic necrosis are listed in Supplementary Table S2. The tumors were independently classified as SCNEC versus LCNEC on H&E slides by two pulmonary pathologists experienced in diagnosis of neuroendocrine carcinomas. Distinction between small and large cell morphology was based primarily on nucleolar features and nuclear:cytoplasmic ratio. Morphologic assessment showed substantial agreement (*κ* = 0.675): both classified seven cases as SCNEC (SCNEC1-7) and four cases as LCNEC (LCNEC1-4). Disagreement was noted for two cases (ANEC1 and ANEC2), each of which showed high nuclear:cytoplasmic ratio yet variably prominent nucleoli. Although distinction between SCNEC and LCNEC for these two cases was not clear based on morphology, all pathologists agreed that each was NEC based on other histopathologic features (organoid architecture, nuclear features, geographic necrosis, and neuroendocrine marker expression). Two mixed tumors (ANEC2 and LCNEC1) demonstrated a component of high-grade IDC-NST in addition to the NEC. Two tumors (SCNEC5 and LCNEC4) exhibited a single-file growth pattern and aberrant E-cadherin expression, consistent with lobular differentiation (Supplementary Fig. S1).

**Table 1:** Clinicopathologic features of neuroendocrine carcinomas.

Case ID	Age (y)	Notting-ham Grade	Tumor Size (cm)	Carcinoma In Situ	Lympho-vascular Invasion	Lymph Node Status	Mixed Component	ER	PR	HER2	Treatment	Follow-Up (m)	Relevant Cancer History
SCNEC1	79	3	1.4	DCIS, F (NE)	-	- (0/1)		-	-	-	Lumpectomy, chemotherapy, brain radiation	Lung and brain metastases; DOD (8)	
SCNEC2	55	3	11.4^a^	-^b^	-^b^	+^c^		-	-	-	Neoadjuvant to palliative chemotherapy, radiation	Lung, liver, and bone metastases; DOD (7)	
SCNEC3	70	3	4	DCIS, F (NE)	+	+^b^		-	-	-	Lumpectomy, chemotherapy, radiation	LFU (3)	Synchronous lung mass (stable after chemotherapy)
SCNEC4	44	3	2	DCIS, F (NE)	-	- (0/21)		-	-	-	Lumpectomy, chemotherapy, radiation	NED (27)	
SCNEC5	65	3	4	LCIS, F (non-NE)	-	- (0/8)		-	-	-	Excision, mastectomy, chemotherapy, endocrine therapy	Liver metastasis (15); LFU	
SCNEC6	57	3	2.5	DCIS (NE)	-	- (0/19)		+	+	-	Mastectomy, chemotherapy	LFU (10)	
SCNEC7	81	3	“large ulcerated wound”	-^b^	-^b^	NP		+	+	-	Palliative chemotherapy, no surgery, endocrine therapy	DOD (4)	Ipsilateral breast cancer 40 years prior (treated with lumpectomy, radiation, chemotherapy)
ANEC1	54	3	3.8	-	-	- (0/4)		-	-	-	Lumpectomy, re-excision, chemotherapy	Chest wall metastasis; DOD (23)	
ANEC2	71	3	5	DCIS, F (non-NE)	+	+ (1/1)	IDC-NST	- (+^d^)	- (+^d^)	-	Mastectomy, chemotherapy, endocrine therapy, excision of metastasis	Multiple metastases; DOD (48)	
LCNEC1	69	3	1.8	DCIS, F (non-NE)	+	+ (4/6)	IDC-NST	- (+^d^)	+ (-^d^)	-	Lumpectomy, chemotherapy, radiation	NED (38)	Synchronous recurrence of base of tongue squamous cell carcinoma
LCNEC2	40	3	5^a^	-^b^	-^b^	+^b^		-	-	-IHC, +FISH	Neoadjuvant chemotherapy (progressed on therapy), no surgery	Liver and bone metastases; AWD (9)	
LCNEC3	38	3	4.3^e^	DCIS, F (NE)	+	+ (2/12)		+	+	- (+^e^)	Neoadjuvant chemotherapy, mastectomy, radiation, chemotherapy, endocrine therapy, BSO	NED (67)	
LCNEC4	74	3	4	DCIS, F (NE)	+	NP		+	+	-	Lumpectomy^f^	Deceased^f^ (8)	

*AWD* alive with disease, *BSO* bilateral salpingo-oophorectomy,  *DCIS* ductal carcinoma in situ, *DOD* died of disease, *F* focal, *FISH* fluorescence in situ hybridization, *IHC* immunohistochemistry, *IDC-NST* invasive ductal carcinoma of no special type, *LCIS* lobular carcinoma in situ, *LFU* lost to follow-up, *NE* neuroendocrine, *NED* no evidence of disease, *NP* not performed.

^a^Based on imaging alone.

^b^Based on core biopsy alone.

^c^Based on fine needle aspiration alone.

^d^In IDC-NST component.

^e^Following neoadjuvant chemotherapy.

^f^Additional information unknown.

**Fig. 2:**
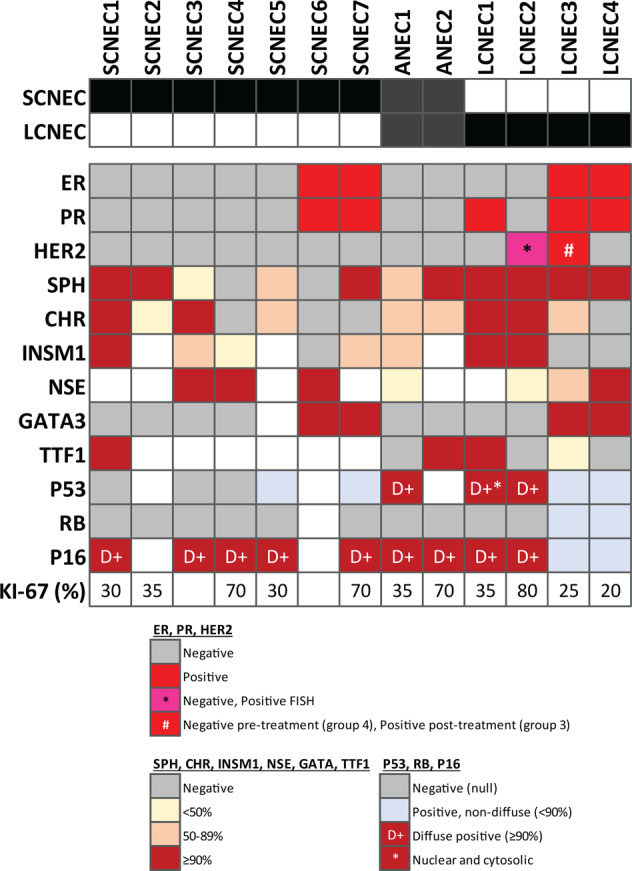
Immunohistochemical profiles of neuroendocrine carcinomas.

By immunohistochemistry, 71% (9/13) of NEC were extensively (≥90% tumor cells) positive for synaptophysin (8/13, 62%) and/or chromogranin (4/13, 31%), with two others (SCNEC5 and ANEC1) showing patchy staining for both neuroendocrine markers (Figs. 1 and 2, Supplementary Table S2). Insulinoma-associated protein 1 (INSM1), an emerging nuclear marker of neuroendocrine differentiation, was expressed in seven NEC, including 4/5 (80%) SCNEC, one ANEC, and 2/4 (50%) LCNEC. By definition, all LCNEC were diffusely positive for synaptophysin (4/4, 100%) and/or chromogranin (2/4, 50%). The majority of SCNEC (4/7, 57%) were diffusely positive for synaptophysin (3/7, 43%) and/or chromogranin (2/7, 29%), with one additional case showing patchy staining for both markers. Of two SCNEC that were synaptophysin and chromogranin negative, both expressed neuron-specific enolase (NSE) diffusely, and one was patchy positive for INSM1. In mixed NEC and IDC-NST tumors, neuroendocrine markers were negative in the IDC-NST component. Nuclear TTF1 was expressed in four tumors (1 SCNEC, 1 ANEC, and 2 LCNEC), all of which had associated ductal carcinoma in situ (DCIS).

### Diagnostic histologic and immunophenotypic features and classification of grade 3 neuroendocrine tumors

**Fig. 3:**
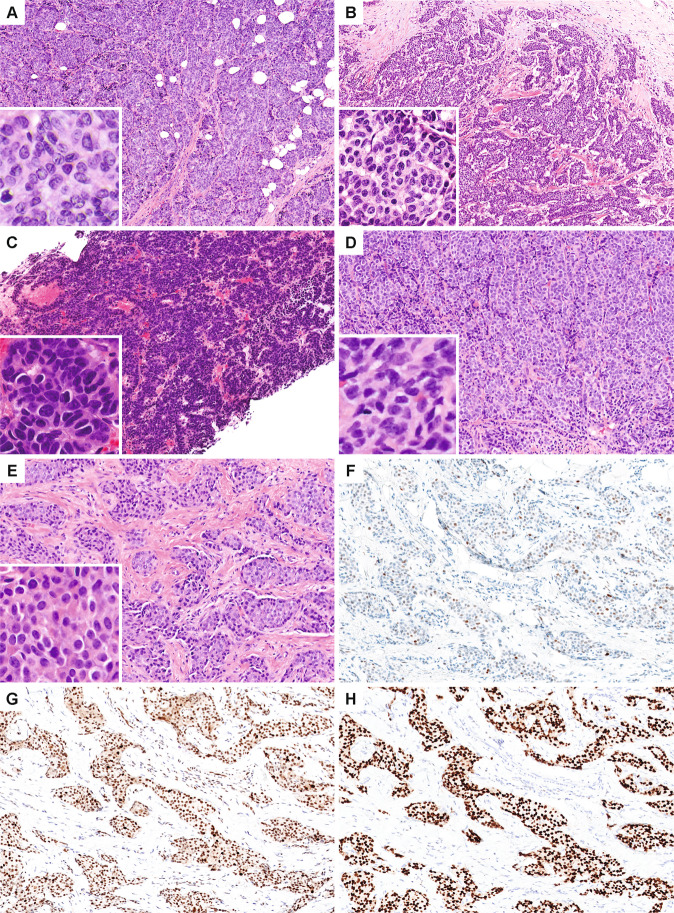
High-grade neuroendocrine neoplasms not meeting criteria for neuroendocrine carcinoma. Nottingham grade 3 breast carcinomas that were diffusely positive for synaptophysin but did not morphologically resemble SCNEC or LCNEC of the lung were designated as neuroendocrine tumors (**A**, NET1; **B**, NET6; **C**, NET7; **D**, NET3; **E**, NET5). These high-grade NET demonstrated intermediate grade nuclei and a high mitotic index. Some exhibited focal glandular architecture (**C**). Immunohistochemical profiles of NET showed a wild-type p53 pattern (**F**), intact RB expression (**G**), and diffusely positive GATA3 (**H**) (NET5 shown).

For purposes of comparison to NEC, eight Nottingham grade 3 NEN with diffuse neuroendocrine morphology and extensive (>90%) synaptophysin expression that did not show characteristic cytomorphologic features of either SCNEC or LCNEC of the lung were identified. In contrast to tumors classified as NEC, these tumors had moderate (not marked) nuclear pleomorphism and lacked necrosis, large prominent nucleoli of LCNEC, nuclear molding, and apoptosis/single-cell necrosis, although mitotic activity was high, resulting in overall Nottingham grade 3 (Fig. 3, Supplementary Table S3, and Supplementary Fig. S2). The tumors were classified as grade 3 NET.

### Clinicopathologic features of neuroendocrine carcinomas

Clinicopathologic features of the 11 pure NEC and 2 mixed NEC/IDC-NST are shown in Table 1. Patient ages ranged from 38 to 81 years (mean 61 years). The majority of tumors (9/13, 69%) were associated with an in situ component that was generally focal, including eight cases with DCIS and one E-cadherin negative SCNEC with lobular carcinoma in situ (LCIS) (SCNEC5). The in situ carcinoma had neuroendocrine differentiation in six cases (6/8 DCIS), with two DCIS and one LCIS being non-neuroendocrine. Lymphovascular invasion (5/13, 38%) and/or lymph node metastasis (6/11, 55%) were identified at presentation in 54% of cases (7/13). Of patients with clinical follow-up, 60% (6/10) developed distant metastases and 50% (5/10) died of disease (mean follow-up 21 months, range 3–67).

In most cases (7/13, 54%), NEC and NEC components of mixed NEC/IDC-NST were triple negative for ER, PR, and HER2, including 71% (5/7) SCNEC and both ANEC. One LCNEC (LCNEC2) was triple negative by immunohistochemistry but HER2 low amplified by fluorescence in situ hybridization (FISH) (ASCO/CAP group 1, HER2/CEN17 ratio 2.1). Another ER+ PR+ LCNEC (LCNEC3) was HER2 negative before neoadjuvant chemotherapy (ASCO/CAP FISH group 4) but HER2 positive (ASCO/CAP FISH group 3) after treatment. Of the two mixed NEC/IDC-NST, both NEC components were ER- (LCNEC1 was PR+) and HER2-, and both IDC-NST components were ER+ and HER2- (Table 1).

Among patients with available treatment information (12/13), all received chemotherapy (Supplementary Table S4); six of seven patients with SCNEC and one of two patients with ANEC received etoposide, an agent standardly used for small cell lung cancer but not included in guidelines for the management of breast cancer^29^.

No statistically significant associations were identified between SCNEC and LCNEC with respect to patient age, tumor size, lymphovascular invasion, lymph node metastasis, biomarker status, or outcomes.

### Genetics of neuroendocrine carcinomas

**Fig. 4:**
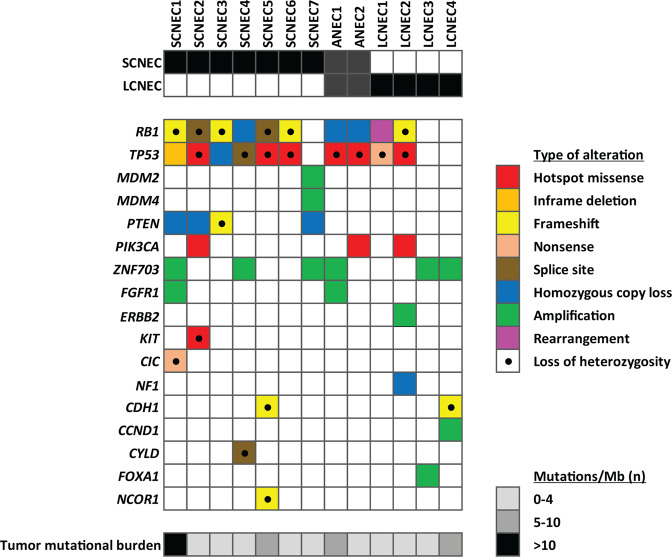
Genetic profiles of neuroendocrine carcinomas.

Results of targeted DNA sequencing are shown in Fig. 4 and Supplementary Tables S5 and S6. The mean target sequencing coverage was 506 (±219) unique reads per target interval (Supplementary Table S7). Tumor mutational burden ranged from <1 to 18 mutations/megabase (median 4). No pathogenic or likely pathogenic germline alterations were identified in any cases.

The most frequent pathogenic aberrations were in *TP53* and *RB1*, which were co-altered in 77% (10/13) of NEC. Of the seven SCNEC, six (86%) exhibited co-alteration of *TP53* and *RB1*. The only SCNEC that lacked *TP53* and *RB1* aberrations (SCNEC7) showed amplifications of *MDM2* and *MDM4*, which are well-known negative regulators of p53^30–32^. Despite the absence of identified *RB1* alteration, this tumor was RB negative by immunohistochemistry (see below). *TP53* and *RB1* co-alteration was also identified in both NEC with ambiguous small versus large cell morphology (ANEC1 and 2) and in 50% (2/4) LCNEC. *TP53*/*RB1* co-alteration was significantly more common in NEC as a group and in SCNEC than in a group of matched grade 3 IDC-NST profiled by UCSF500 assay (7/45, 16%) (p < 0.001 and p = 0.001, respectively). Similar results were found when all NEC or SCNEC were compared to grade 3 IDC-NST in the large publicly available METABRIC dataset, in which *TP53*/*RB1* co-alteration was reported in only 3% (35/1009) tumors (*p* < 0.001, *p* < 0.001, and *p* = 0.007, respectively) (Supplementary Fig. S3).

Pathogenic phosphoinositide (PI)-3 kinase pathway aberrations were identified in 46% (6/13) NEC. These included homozygous *PTEN* loss in 57% (4/7) SCNEC but not in any LCNEC or ANEC, and activating hotspot *PIK3CA* mutations in 3 tumors (1 SCNEC, 1 ANEC, 1 LCNEC). *ZNF703* amplifications were identified in 43% (6/13) NEC, half of which were triple negative, and included 43% (3/7) SCNEC, 50% (2/4) LCNEC, and 1 ANEC. *FGFR1* was co-amplified with *ZNF703* in two cases (1 SCNEC and 1 ANEC), with equivocal *FGFR1* amplification in a third (SCNEC7) (Supplementary Table S6). One SCNEC and one LCNEC each had a *CDH1* truncating mutation with loss of heterozygosity (LOH), consistent with the morphologic and immunophenotypic (E-cadherin loss) impression of lobular differentiation in these tumors (Fig. 4 and Supplementary Fig. S1).

Copy number analysis showed numerous chromosomal gains and losses. Recurrent gains were detected in distal 8q (9/11, 82%) and proximal 1q (7/11, 64%) and recurrent losses were observed in interstitial 22q (10/11, 91%), proximal 13q (8/11, 73%), interstitial 16q (8/11, 73%), distal 8p (8/11, 73%), and proximal 15q (6/11, 55%). No statistically significant copy number differences were identified between SCNEC and LCNEC.

### Immunohistochemical expression of RB, p16, p53, and other markers in neuroendocrine carcinomas with comparison to grade 3 neuroendocrine tumors

**Fig. 5:**
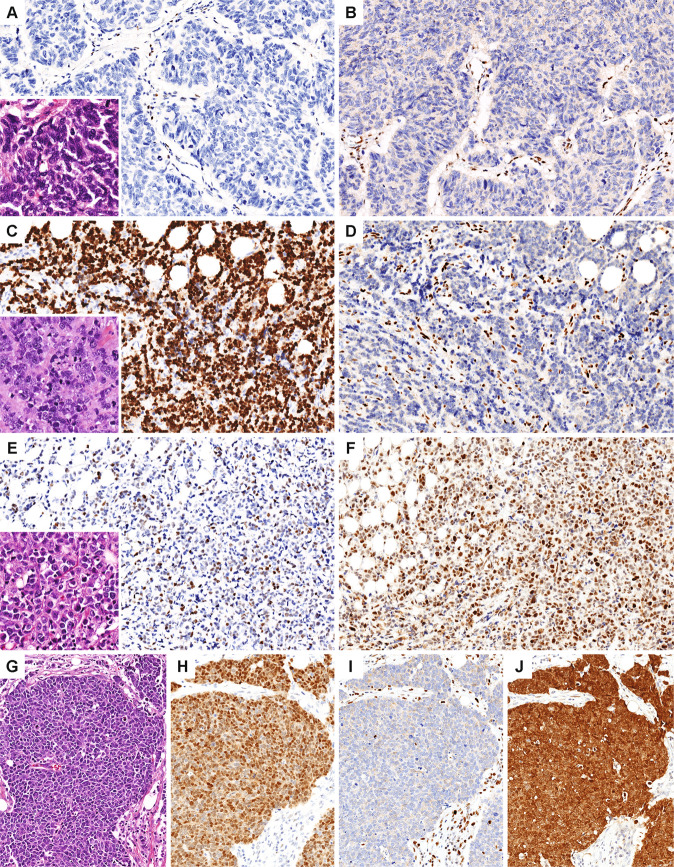
Aberrant immunohistochemical expression patterns of RB, p53, and p16 in neuroendocrine carcinomas. Co-alteration of RB and p53 was frequent in NEC, such as a null p53 staining pattern (**A**) and negative RB expression (**B**) in SCNEC1, and diffusely positive (overexpressed) p53 staining pattern (**C**) with negative RB expression (**D**) in ANEC1. A subset of LCNEC and all non-NEC exhibited wild-type p53 (**E**) and intact RB expression (**F**) (LCNEC4). **G-J** LCNEC1 demonstrated an unusual aberrant nuclear and cytoplasmic staining pattern for p53 (**H**) with negative RB expression (**I**). p16 was diffusely positive in all tumors with RB loss (**J**).

To further explore RB and p53 pathway inactivation in NEC, immunohistochemical stains for RB, p16, and p53 were performed on tumors with available tissue (Table 2, Figs. 2 and 5). All NEC or NEC components of mixed NEC/IDC-NST harboring *RB1* alterations (including frameshift and splice site mutations, deep deletions, and genomic rearrangement) were RB negative by immunohistochemistry, including all SCNEC (6/6) and ANEC (2/2), and 2/4 LCNEC. Notably, the only SCNEC lacking *RB1* alteration (SCNEC7) was RB negative by immunohistochemistry. Both LCNEC without *RB1* alterations showed intact RB staining. Diffuse p16 overexpression was seen in 82% (9/11) NEC (5/5 SCNEC, 2/2 ANEC, 2/4 LCNEC) and was exclusively associated with RB loss. An aberrant (null type or diffuse positive) p53 staining pattern was seen in 6/10 (60%) NEC, including 3/5 SCNEC, one ANEC, and 2/4 LCNEC. Six of seven (86%) tumors with *TP53* alterations demonstrated aberrant p53 expression, whereas none of the three tumors without *TP53* alterations had aberrant p53 expression. Negative p53 staining was associated with large deletion, focal homozygous deletion, and splice site mutation in *TP53* (three SCNEC), whereas diffuse p53 staining was associated with missense mutations in two tumors and with the p.R342* nonsense mutation (LCNEC1), the latter of which showed nuclear as well as cytoplasmic staining^33,34^. Of note, *TP53* p.G266E mutation has been previously shown to be associated with a non-diffuse staining pattern, as was seen in SCNEC5^33^.

**Table 2:** RB and p53 alterations in neuroendocrine carcinomas.

Case ID	*TP53* alteration	p53 IHC	*RB1* alteration	RB IHC
**SCNEC1**	p.Y126_R158del	−	p.D394fs	−
**SCNEC2**	p.C238F	NP	c.2212-1G>C	−
**SCNEC3**	Deep deletion	−	p.K202fs	−
**SCNEC4**	c.993+1G>A	−	Deep deletion	−
**SCNEC5**	p.G266E	+	c.2107-1G>C	−
**SCNEC6**	p.R213L	NP	p.T197fs	NP
**SCNEC7**	None	+	NP	−
**ANEC1**	p.M133K	++	Deep deletion	−
**ANEC2**	p.R248W	NP	Deep deletion	−
**LCNEC1**	p.R342*	++^a^	Rearrangement	−
**LCNEC2**	p.Y163C	++	p.Y325fs	−
**LCNEC3**	None	+	None	+
**LCNEC4**	None	+	None	+

−Negative (null); +Positive, non-diffuse (<90%); ++Positive, diffuse (≥90%).

*IHC* immunohistochemistry, *NP* not performed.

^a^Nuclear and cytoplasmic staining.

For comparison to NEC, immunohistochemical expression of RB, p53, and p16 was also assessed in the group of grade 3 NET (*n* = 8), as well as in two grade 3 IDC-NED and one grade 3 ILC-NED (Fig. 3 and Supplementary Tables S3 and S8). In contrast to the NEC, none of the other grade 3 cancers showed aberrant RB or p53 expression patterns (RB loss in 83% NEC vs 0% grade 3 NET, *p* = 0.001; aberrant p53 in 60% NEC vs 0% grade 3 NET, *p* = 0.013), and none were diffusely p16 positive (82% NEC vs 0% grade 3 NET, *p* = 0.002). The results were also statistically significant when only SCNEC were considered in the analysis (RB loss in 100% SCNEC vs 0% grade 3 NET, *p* < 0.001; aberrant p53 in 50% SCNEC vs 0% grade 3 NET, *p* = 0.035; diffuse p16 in 100% SCNEC vs 0% grade 3 NET, *p* = 0.001). No statistically significant differences in RB, p53, or p16 expression patterns were identified between LCNEC and grade 3 NET (RB loss, aberrant p53, and diffuse p16 in 50% LCNEC vs 0% grade 3 NET, *p* = 0.091, *p* = 0.091, and *p* = 0.109, respectively). NEC and SCNEC were also more frequently RB negative than a control group of 95 grade 3 IDC-NST enriched for triple negative carcinomas (83% NEC and 100% SCNEC vs 38% IDC-NST, *p* = 0.004 each).

NEC as a group and SCNEC were each more likely than grade 3 NET to be ER negative (69% NEC and 71% SCNEC vs 0% grade 3 NET; *p* = 0.005 and *p* = 0.007, respectively) and GATA3 negative (67% NEC and 67% SCNEC vs 0% grade 3 NET, *p* = 0.013 and *p* = 0.021, respectively) (Supplementary Tables S3 and S8). No statistically significant differences in ER or GATA3 expression were identified between LCNEC and grade 3 NET (ER negative in 50% LCNEC vs 0% grade 3 NET, *p* = 0.091; GATA3 negative in 50% LCNEC vs 0% grade 3 NET, *p* = 0.109). No statistically significant differences in Ki-67 proliferation index were identified between any of the groups (Fig. 2 and Supplementary Table S8). All tested NEC and other grade 3 NEN expressed ATRX (*n* = 12 NEC, *n* = 10 other NEN) and were negative for high-risk papillomavirus by in situ hybridization (*n* = 11 NEC, *n* = 9 other NEN) (Supplementary Table S8).

### Genetic and immunophenotypic analysis of neuroendocrine and invasive ductal carcinoma components of mixed tumors

**Fig. 6:**
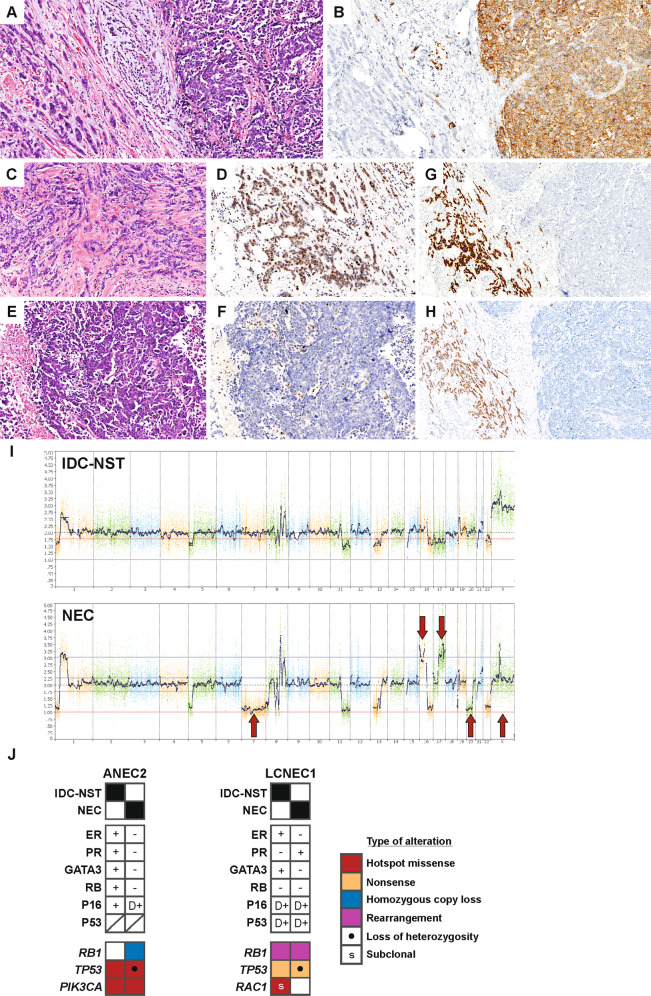
Mixed neuroendocrine carcinoma and invasive ductal carcinoma of no special type. ANEC2 was comprised of mixed components of NEC and IDC-NST (**A** *left*, IDC-NST; *right,* NEC). **B** Synaptophysin highlights the NEC component. **C**, **D** The IDC-NST component shows intact RB expression (**D**); **E**, **F** The NEC component is RB negative (**F**). **G** ER is diffusely positive in IDC-NST (*left*) and negative in the NEC component (*right*). **H** GATA3 expression mirrors ER. **I** Chromosomal copy number plots reveal multiple gains and losses that are shared between the IDC-NST and NEC components, with additional alterations only in NEC (*red arrows*). **J** Immunohistochemical and genetic profiles of the separately analyzed components of ANEC2 and LCNEC1, summarizing features that are shared and unique between the components.

The neuroendocrine and IDC-NST components of two mixed NEC/IDC-NST tumors (ANEC2 and LCNEC1) were separately analyzed by immunohistochemistry and targeted DNA sequencing (Fig. 6 and Supplementary Fig. S4).

In ANEC2, the NEC and IDC-NST components shared hotspot *PIK3CA* and *TP53* mutations and numerous chromosomal copy number changes, consistent with shared clonality between the components. LOH of the *TP53* allele and focal homozygous deletion of *RB1* exon 1 were exclusive to the NEC component. Consistent with this, RB protein loss and diffuse p16 expression were also restricted to the NEC component. Whereas the IDC-NST component expressed ER and GATA3, these markers were negative in the NEC areas (Fig. 6).

The LCNEC and IDC-NST components of LCNEC1 shared a duplication involving exons 3–25 of *RB1* and a *TP53* nonsense mutation, as well as numerous chromosomal copy number changes, again consistent with shared clonality between the components. *TP53* LOH was detected only in the NEC component. By immunohistochemistry, RB and p53 were aberrant in both components. The IDC-NST component was ER+ PR- and expressed GATA3, whereas the NEC component showed the converse immunoprofile (ER- PR+ and GATA3 negative) (Fig. 6 and Supplementary Fig. S4).

## Discussion

Small cell lung carcinoma (SCLC), the prototypical poorly-differentiated NEC, demonstrates near-universal biallelic inactivation of tumor suppressor genes *TP53* and *RB1*^35^, and frequent *TP53*/*RB1* co-alteration has also been identified in small cell/poorly-differentiated NEC of the pancreas, prostate, bladder, and colon/rectum, and in Merkel cell carcinoma^36–42^. In contrast to SCLC, LCNEC of the lung is genetically more heterogeneous, with *TP53*/*RB1* co-inactivation only in a subset of tumors, while those lacking these alterations harbor mutations that are more frequently seen in pulmonary adenocarcinomas^43,44^. In contrast to NEC, *TP53* and *RB1* alterations are absent or exceedingly rare in extramammary NET (including G3 NET)^12,45–49^, which instead have mutations in chromatin remodeling genes *MEN1* (lung and pancreas), *DAXX/ATRX* (pancreas), the mTOR pathway (pancreas), and *CDNK1B* (small intestine)^45–51^. In comparison to extramammary NEN, a paucity of data exists for breast tumors with neuroendocrine differentiation.

SCNEC has been recognized as an aggressive type of breast cancer for many years, although most of the literature consists of case reports and small series due to the rarity of these tumors. Histochemical and ultrastructural features of SCNEC were first reported nearly two decades ago in a series of four cases, including the identification of in situ carcinoma supporting primary breast origin^52^. A subsequent series detailed the morphologic features and expanded immunohistochemical findings of nine breast SCNEC, including four that were mixed with non-NEC components^6,9^. The largest series to date comprised 19 SCNEC and identified *TP53* somatic mutations in 6/8 and *PIK3CA* mutations in 3/9 cases using a 47-gene NGS panel^53^. No *RB1* mutations were reported, and *RB1* copy number and RB immunohistochemistry were not assessed. Although limited by the number of cases, our study offers a comprehensive characterization of the molecular landscape of SCNEC. Akin to SCNEC of the lung and other anatomic sites^35–42^, we identified for the first time near-universal *TP53* and *RB1* co-alterations in our cohort of breast SCNEC, with the sole SCNEC that lacked these alterations being RB negative by immunohistochemistry and showing p53 pathway aberrations (*MDM2* and *MDM4* amplifications) that are known negative regulators of p53^30–32^. These findings suggest that co-inactivation of *TP53* and *RB1* may be important for the small cell phenotype in the breast, as at other sites^35–42^. We speculate that methodological differences, notably including the detection of deep deletions, splice site mutations, and a genomic rearrangement, rather than only coding mutations, in our series, may help explain the discrepancy between the mutation prevalence in our study and the prior study that used a small targeted gene panel^53^.

LCNEC of the breast as defined by the most recent WHO classification is thought to be exceedingly rare and remains largely uncharacterized in terms of molecular features and clinical behavior. Although limited by a small number of cases, we show here that strictly defined LCNEC appear to be genetically heterogeneous, with some but not all harboring *TP53*/*RB1* co-alteration. Two NEC with ambiguous or mixed small versus large cell features (ANEC) in our study also had *TP53*/*RB1* co-alteration. The genetics of breast LCNEC thus appear to mirror the genetic heterogeneity described in pulmonary LCNEC, where some LCNEC are SCNEC-like and others are not^44^. Whether LCNEC of the breast with and without p53/Rb co-alterations are clinically distinct from one another and/or from other high-grade NEN of the breast will require larger follow-up studies of patients with these rare tumors.

SCNEC with mixed NEC and non-NEC components have been previously reported^6^. We describe here the genetic and immunohistochemical analysis of a mixed ANEC/IDC-NST and a mixed LCNEC/IDC-NST, both of which were also associated with non-neuroendocrine DCIS. Genetic analysis of separate invasive NEC and IDC-NST areas of these mixed tumors confirms a shared clonality between the components and raises the possibility that NEC could arise as a secondary event from ductal carcinoma in the breast rather than *de novo* from a committed neuroendocrine progenitor cell. Indeed, a native neuroendocrine progenitor cell has not been identified in the breast^11,54,55^. We also show that LOH of a shared *TP53* mutation was restricted to the NEC components of both mixed tumors, while deep deletion of *RB1* was exclusive to the NEC component in one, again suggesting a role for *TP53*/*RB1* co-inactivation in the pathogenesis of the NEC phenotype in the breast, including at least a subset of LCNEC. On the other hand, *TP53*/*RB1* co-inactivation is by no means exclusive to NEC in the breast and is also found in a subset of non-NEC basal-type triple negative and luminal B breast cancers^56–59^.

We identified amplification of *ZNF703* in 46% of NEC (three SCNEC, one ANEC, and two LCNEC), half of which were triple negative and half of which were ER positive (one LCNEC was triple positive). *FGFR1* was co-amplified in two of the triple negative tumors. *ZNF703* encodes a zinc finger protein and estrogen-responsive transcriptional cofactor, which appears to be preferentially amplified in luminal B breast cancers and is associated with high proliferation and high histologic grade^60,61^. *FGFR1* amplification is also common in ER-positive carcinomas and has been associated with increased grade and proliferation index^62,63^. The significance of *ZNF703* amplifications in triple negative NEC is not known. However, we note that the IDC-NST components of both mixed NEC/IDC-NST tumors in our series were ER positive, whereas the clonally related NEC components were ER negative. Together, this raises speculation that at least some ER negative SCNEC and LCNEC may be intrinsic luminal B tumors that have lost ER expression during tumor progression, which could explain the enrichment of luminal-type alterations in these tumors. Intrinsic molecular subtyping would help to conclusively address this question in the future.

Inactivating *CDH1* mutations were identified in one SCNEC and one LCNEC and corresponded to aberrant E-cadherin expression by immunohistochemistry, supportive of lobular differentiation of these tumors. Although neuroendocrine differentiation has been reported in rare lobular carcinomas, the identification of the lobular phenotype in SCNEC and LCNEC is, to the extent of our knowledge, a novel finding^64–66^.

Most previous studies of breast tumors with neuroendocrine differentiation report an association with luminal subtypes and predominantly include NET and IDC-NED using the current taxonomy; relatively few grade 3 cancers have been studied. Ang et al. identified recurrent *PIK3CA* (3/15 cases) and FGFR (2/15) alterations in their series, which included only three grade 3 cancers, two with no detected alterations and one which had a pathogenic *HRAS* mutation^64^. Marchiò et al. reported recurrent alterations in *GATA3*, *FOXA1*, *TBX3*, *ARID1A*, *PIK3CA*, *AKT1*, and *CDH1* in a series of IDC-NED, mucinous, and solid papillary carcinomas, which included only three grade 3 cancers; *FOXA1*, *CDH1*, *AKT1*, and *KMT2C* alterations were seen in one of the latter^65^. Neither of these studies appeared to include SCNEC or LCNEC. A study by Lavigne et al. using 2012 (4th edition) WHO terminology included 15 grade 3 cancers, with *TP53* mutations identified in two “poorly-differentiated neuroendocrine carcinomas” and one grade 2 NET^67^. *PIK3CA* mutations were identified in grade 2 and 3 NET. Wei et al. recently reported a genetic analysis of 11 NEN classified as NEC and found no *TP53* or *RB1* alterations in any of the tumors, although other genes in the p53 and RB pathways were mutated at 18% and 27%, respectively^68^. Neither of these latter two studies specified whether the analyzed tumors were SCNEC, LCNEC, or neither. Taken together, the genetics of NEC in our study are clearly distinct from those of the NET and IBC-NED reported in prior studies, supporting the separate classification of NEC. With respect to NEC included in the study by Wei et al., differences in tumor classification are likely to be at least partly responsible for apparent discrepancies from our results.

The 5th edition of the WHO classification of breast tumors defines NET as NEN with >90% neuroendocrine morphology and “extensive” neuroendocrine expression by immunohistochemistry. Defined as such, NET are *usually* Nottingham grade 1–2, and such tumors can be readily distinguished from NEC, which are high-grade tumors that resemble pulmonary NEC^2,5,11^. However, NEN with intermediate nuclear grade and high mitotic activity (Nottingham grade 3) that do not resemble pulmonary NEC morphologically are occasionally encountered in practice, although more specific diagnostic criteria to distinguish these tumors from NEC are lacking. Classification of such grade 3 NEN tumors using the WHO system is nebulous. Indeed, they are not excluded from the NET definition and can be best classified as grade 3 NET if adhering strictly to the diagnostic criteria^1,11^. On the other hand, it is unclear if some authors interpret the WHO classification such that all grade 3 NEN are NEC and all grade 1–2 NEN are NET^68,69^. In the enteropancreatic system, it has been established that NET with well-differentiated morphology but high mitotic activity and/or Ki-67 index (grade 3 NET) are genetically and clinically distinct from NEC. An analogous dichotomy of grade 3 NEN has not been established in the breast. In this study, we classified a group of uncommon Nottingham grade 3 NEN that did not morphologically resemble NEC of the lung as grade 3 NET and compared them to our cohort of NEC. None of the grade 3 NET showed aberrant RB or p53 staining patterns or diffuse p16 expression, in contrast to the high frequency seen in the NEC, including both SCNEC and LCNEC. We note that immunohistochemical expression of p53 and especially RB reliably correlated with underlying *RB1* and *TP53* genetic alterations in this study and others^33^. Strong diffuse overexpression of the cyclin-dependent kinase inhibitor p16 also correlated exclusively with RB alteration, presumably due to the known negative feedback regulation of p16 by RB, and was only seen in NEC^70,71^. The immunohistochemical findings thus suggest differences in the underlying genetics and biology of NEC compared to tumors classified as grade 3 NET in our study. Significantly higher frequencies of ER and GATA3 expression in grade 3 NET further support an alternate phenotype from NEC. The small number of cases in our series limits any meaningful comparative outcome analysis. However, we note that 60% of patients with NEC developed distant metastases and 50% died of disease (mean follow-up 21 months, range 3–67), whereas no such adverse events were found in patients with grade 3 NET (mean follow-up 32 months, range 3–88). Overall, additional molecular and clinical outcome studies with larger numbers of carefully classified tumors will be essential to determine whether tumors with features of grade 3 NET are biologically distinct from NEC, or whether they should be considered along the spectrum of a heterogeneous group of LCNEC without p53/RB alterations. In this context and given the difficulty in morphologically distinguishing some LCNEC from grade 3 NET, we can speculate that classification of grade 3 NEN based on p53/RB alteration rather than morphology may be clinically relevant.

Our findings raise important questions regarding whether SCNEC of the breast should be managed distinctly from other high-grade breast carcinomas, especially triple-negative carcinomas. We have shown that SCNEC of the breast is convergent genetically, as well as histologically, with SCNEC of the lung, with frequent concurrent loss of p53 and RB. However, we also uncovered clonal origin of mixed NEC/IDC-NST, suggesting that SCNEC may genetically descend from an earlier IDC clone, rather than a distinct progenitor cell. Numerous case reports in the literature reflect the current uncertainty around optimal management of mammary SCNEC, including frequent reports of etoposide-based regimens that are used for SCNEC of the lung and not standardly for breast cancer^29,72–77^. In our series, the majority of patients with SCNEC were treated with etoposide (6/7, 86%), indicating that the diagnosis can influence medical oncologists’ choice of therapy, even in the absence of specific guidelines.

In summary, we have provided a comprehensive genetic characterization of carefully classified NEC of the breast and show that p53 and RB pathway co-alterations are highly prevalent in SCNEC and a subset of LCNEC, similar to their counterparts in the lung^35,43,44^. Genetic and immunophenotypic analysis of paired NEC and IDC-NST components of mixed tumors confirms their shared clonality and suggests that the NEC phenotype could arise from ductal carcinoma in the breast. Classification of uncommon grade 3 NEN without classic features of NEC is problematic in the most recent WHO classification, and our data suggest that these tumors may be biologically distinct from NEC, or, alternatively, that they fall along a spectrum of heterogeneous LCNEC that lack p53 and RB alterations. The clinical significance of recognizing grade 3 NEN with and without p53/RB alterations awaits further study.

## Supplementary information

Supplementary Tables

Supplementary Figures

## Data Availability

Data generated and analyzed during the current study are available from the corresponding author on reasonable request.
