# Correction to: Genetic and immunohistochemical profiling of small cell and large cell neuroendocrine carcinomas of the breast

**DOI:** 10.1038/s41379-022-01099-3

**Published:** 2022-06-13

**Authors:** Gregory R. Bean, Saleh Najjar, Sandra J. Shin, Elizabeth M. Hosfield, Jennifer L. Caswell-Jin, Anatoly Urisman, Kirk D. Jones, Yunn-Yi Chen, Gregor Krings

**Affiliations:** 1 Department of Pathology, Stanford University School of Medicine, Stanford, CA USA; 2 Department of Pathology and Laboratory Medicine, Albany Medical College, Albany, NY USA; 3 Department of Pathology, Kaiser Permanente San Francisco Medical Center, San Francisco, CA USA; 4 Department of Medicine, Division of Oncology, Stanford University School of Medicine, Stanford, CA USA; 5 Department of Pathology, University of California San Francisco, San Francisco, CA USA

**Keywords:** Pathology, Breast cancer, Translational research, Cancer genomics, Oncogenes

Correction to: *Modern Pathology* 10.1038/s41379-022-01090-y, published online 19 May 2022

The original version of this article unfortunately contained mistakes. The errors are:

1. in Table 2: For LCNEC2, p53 IHC should be ++, not +

**Table 2:** RB and p53 alterations in neuroendocrine carcinomas.

Case ID	*TP53* alteration	p53 IHC	*RB1* alteration	RB IHC
SCNEC1	p.Y126_R158del	−	p.D394fs	−
SCNEC2	p.C238F	NP	c.2212-1G>C	−
SCNEC3	Deep deletion	−	p.K202fs	−
SCNEC4	c.993+1G>A	−	Deep deletion	−
SCNEC5	p.G266E	+	c.2107-1G>C	−
SCNEC6	p.R213L	NP	p.T197fs	NP
SCNEC7	None	+	NP	−
ANEC1	p.M133K	++	Deep deletion	−
ANEC2	p.R248W	NP	Deep deletion	−
LCNEC1	p.R342*	++^a^	Rearrangement	−
LCNEC2	p.Y163C	++	p.Y325fs	−
LCNEC3	None	+	None	+
LCNEC4	None	+	None	+

−Negative (null); +Positive, non-diffuse (<90%); ++Positive, diffuse (≥90%).

*IHC* immunohistochemistry, *NP* not performed.

^a^Nuclear and cytoplasmic staining.
2. in Figure 4: the type of RB mutation for SCNEC6 is incorrect (box should be yellow not peach). The corrected table and figure can be found below. The original article has been corrected.

