# Supplementary material for: Genetic and immunohistochemical profiling of small cell and large cell neuroendocrine carcinomas of the breast
Source: Mod Pathol. 2022 May 19;35(10):1349–61. doi: 10.1038/s41379-022-01090-y (PMC9514991; doi:10.1038/s41379-022-01090-y)
Supplement: Supplementary file 1 — Supplementary Tables [file 41379_2022_1090_MOESM1_ESM.docx]

**Supplementary Table S1.** UCSF500 gene list.

| ABL1 | ABL2 | ACVR1 | ACVR1B | AJUBA | AKT1 | AKT2 | AKT3 | ALK | APC | APOBEC3G |
| --- | --- | --- | --- | --- | --- | --- | --- | --- | --- | --- |
| AR | ARAF | ARFRP1 | ARHGAP35 | ARID1A | ARID1B | ARID2 | ARID5B | ASH2L | ASXL1 | ASXL2 |
| ATF1 | ATM | ATR | ATRX | AURKA | AURKB | AXIN1 | AXIN2 | AXL | BAP1 | BARD1 |
| BCL2 | BCL2A1 | BCL2L1 | BCL2L12 | BCL2L2 | BCL6 | BCOR | BCORL1 | BLM | BRAF | BRCA1 |
| BRCA2 | BRD4 | BRIP1 | BTG1 | BTK | CALR | CARD11 | CBFB | CBL | CBLB | CCND1 |
| CCND2 | CCND3 | CCNE1 | CD79A | CD79B | CD274 | CDC42 | CDC73 | CDH1 | CDK12 | CDK4 |
| CDK6 | CDK8 | CDKN1A | CDKN1B | CDKN2A | CDKN2B | CDKN2C | CEBPA | CHD1 | CHD2 | CHD4 |
| CHD5 | CHEK1 | CHEK2 | CIC | CLDN18 | CNOT3 | COL1A1 | COL2A1 | CRCT1 | CREB1 | CREBBP |
| CRKL | CSF1R | CSF3R | CTCF | CTNNA1 | CTNNB1 | CUL3 | CUX1 | CYLD | CXCR4 | DCC |
| DDIT3 | DDR2 | DDX3X | DDX41 | DGKH | DICER1 | DIS3 | DNAJB1 | DNMT3A | DOT1L | DUSP2 |
| DUSP4 | DUSP6 | DYNC1I1 | EBF1 | EDNRB | EGFR | EGR1 | EIF1AX | ELF3 | EMSY (C11orf30) | EP300 |
| EPCAM | EPHA2 | EPHA3 | EPHA5 | EPHA7 | EPHB1 | EPOR | ERBB2 | ERBB3 | ERBB4 | ERCC1 |
| ERCC2 | ERG | ERRFI1 | ESPL1 | ESR1 | ESR2 | ETS1 | ETV6 | EWSR1 | EZH1 | EZH2 |
| FAM123B (WTX) | FAM46C | FANCA | FANCC | FANCE | FANCF | FANCG | FANCL | FAT1 | FAT3 | FBXW7 |
| FGF10 | FGF14 | FGF19 | FGF23 | FGF3 | FGF4 | FGF6 | FGFR1 | FGFR2 | FGFR3 | FGFR4 |
| FH | FLCN | FLT1 | FLT3 | FLT4 | FOXA1 | FOXL2 | FOXO1 | FOXP1 | FRS2 | FUBP1 |
| FUS | FYN | GAB2 | GATA1 | GATA2 | GATA3 | GLI1 | GLI2 | GNA11 | GNA13 | GNAQ |
| GNAS | GPC3 | GPR124  (ADGRA2) | GRIN2A | GRM3 | GSK3B | H3F3A | H3F3B | HDAC4 | HDAC9 | HEY1 |
| HGF | HIF1A | HIST1H3B | HMGA2 | HNF1A | HOXB13 | HRAS | HSPA2 | HSPA5 | HSP90AB1 | ID3 |
| IDH1 | IDH2 | IGF1R | IGF2 | IGF2R | IKBKE | IKZF1 | IKZF2 | IKZF3 | IL2RB | IL7R |
| INHBA | INPP4B | IPMK | IRF4 | IRS2 | JAK1 | JAK2 | JAK3 | JAZF1 | KAT6A (MYST3) | KDM5A |
| KDM5C | KDM6A | KDR | KEAP1 | KIT | KLF4 | KLHL6 | KMT2A | KMT2B | KMT2D | KNSTRN |
| KRAS | LEF1 | LIFR | LRP1B | LZTR1 | MALAT1 | MAML2 | MAP2K1 | MAP2K2 | MAP2K4 |  |
| MAP3K1 | MAP3K2 | MAP3K5 | MAP3K7 | MAP3K9 | MAPK1 | MCL1 | MDM2 | MDM4 | MED12 | MEF2B |
| MEN1 | MET | MGA | MGMT | MITF | MLH1 | MLH3 | MPL | MRE11A | MSH2 | MSH3 |
| MSH6 | MTOR | MUTYH | MYB | MYBL1 | MYC | MYCL1 | MYCN | MYD88 | MYH9 | NAV3 |
| NBN | NCKAP5 | NCOA2 | NCOA3 | NCOR1 | NF1 | NF2 | NFE2L2 | NFKBIA | NFKBIE | NIPBL |
| NKX2-1 | NOTCH1 | NOTCH3 | NPM1 | NRAS | NSD1 | NSD2 | NT5C2 | NTRK1 | NTRK2 | NTRK3 |
| NUP93 | NUTM1 | OR5L1 | PAK1 | PAK3 | PALB2 | PARK2 | PAX3 | PAX5 | PAX7 | PAX8 |
| PBRM1 | PDCD1LG2 | PDGFB | PDGFRA | PDGFRB | PDK1 | PHF6 | PHOX2B | PIK3CA | PIK3CG | PIK3R1 |
| PIK3R2 | PLAG1 | PLCB4 | PMS1 | POLD1 | POLE | POLQ | POT1 | POU3F2 | PPM1D | PPP2R1A |
| PPP6C | PRDM1 | PREX2 | PRKACA | PRKAG2 | PRKAR1A | PRKCA | PRKCH | PRKDC | PTCH1 | PTCH2 |
| PTEN | PTK2B | PTPN1 | PTPN11 | PTPRB | PTPRD | PTPRK | PTPRT | RAC1 | RAD21 | RAD50 |
| RAD51 | RAD51C | RAD51D | RAF1 | RARA | RASA1 | RASA2 | RB1 | RBM10 | REL | RELA |
| RET | RHEB | RHOA | RICTOR | RIT1 | RNF43 | ROBO1 | ROS1 | RPL10 | RPTOR | RRAGC |
| RRAS | RRAS2 | RSPO2 | RSPO3 | RUNX1 | RUNX1T1 | SDHB | SDHD | SETBP1 | SETD2 | SF3B1 |
| SH2B3 | SHH | SIN3A | SLIT2 | SLITRK6 | SMAD2 | SMAD3 | SMAD4 | SMARCA2 | SMARCA4 | SMARCB1 |
| SMC1A | SMC3 | SMO | SNCAIP | SOCS1 | SOS1 | SOS2 | SOX9 | SOX10 | SOX2 | SPEN |
| SPOP | SPRED1 | SPRY1 | SPRY2 | SPRY4 | SPTA1 | SRC | SRSF2 | SS18 | STAG2 | STAT3 |
| STAT4 | STAT6 | STK11 | SUFU | SYK | SYNE1 | TADA1 | TBX3 | TCEB1 | TCF7L2 | TERT |
| TET2 | TFE3 | TFEB | TGFBR2 | TLR4 | TNFAIP3 | TNFRSF14 | TOP1 | TOP2A | TMPRSS2 | TP53 |
| TRAF3 | TRAF7 | TRIM28 | TSC1 | TSC2 | TSHR | TSHZ2 | TSHZ3 | TSLP | TTYH1 | TYK2 |
| U2AF1 | USP7 | VEGFA | VHL | WISP3 | WRN | WT1 | XBP1 | XPO1 | YAP1 | YWHAE |
| ZBTB20 | ZFHX3 | ZMYM3 | ­ZNF217 | ZNF703 | ZNFHX4 | ZRSR2 |  |  |  |  |

**Supplementary Table S2.** Cytomorphologic features and neuroendocrine marker immunohistochemistry of neuroendocrine carcinomas.

| **Case ID** | **Type** | **Nuclear:**  **cytoplasmic Ratio** | **Nuclear Features** | **Nucleoli** | **Cytoplasm** | **Growth Pattern** | **Geographic Necrosis** | **SYN** | **CHR** | **INSM1** | **Other** |
| --- | --- | --- | --- | --- | --- | --- | --- | --- | --- | --- | --- |
| **SCNEC1** | Small cell | High | Spindled, speckled to vesicular chromatin | Indistinct | Indistinct | Circumscribed, trabecular to ribbon-like | + | +++ | +++ | +++ | TTF1+ |
| **SCNEC2** | Small cell | High | Oval, speckled chromatin, crush artifact | Indistinct | Indistinct | Infiltrative, organoid nests | + | +++ | + | NP | NP |
| **SCNEC3** | Small cell | High | Oval, speckled chromatin | Indistinct | Indistinct | Expansive, organoid nests | + | + | +++ | ++ | NSE+ |
| **SCNEC4** | Small cell | High | Oval/polygonal, speckled chromatin, molding | Indistinct | Indistinct to eosinophilic granular | Expansive, solid | + | - | - | + | NSE+ |
| **SCNEC5** | Small cell | High | Oval, speckled chromatin | Indistinct | Indistinct | Infiltrative, sheets and cords | - | ++ | ++ | NP | NP |
| **SCNEC6** | Small cell | High | Oval, speckled chromatin, crush artifact | Indistinct | Indistinct | Infiltrative, organoid nests and cords | - | - | - | - | NSE+ |
| **SCNEC7** | Small cell | High | Oval, speckled chromatin | Indistinct | Indistinct to eosinophilic granular | Infiltrative, organoid nests | + | +++ | - | ++ | NP |
| **ANEC1** | Ambiguous | High | Oval/polygonal, speckled to vesicular chromatin, focal molding | Prominent | Indistinct to pale granular | Expansive, sheets to organoid nests | + | ++ | ++ | ++ | NSE+ TTF1- |
| **ANEC2** | Ambiguous | High | Oval, vesicular chromatin, occasionally multinucleated | Pinpoint to prominent | Indistinct to eosinophilic granular | Expansive, solid, organoid | + | +++ | ++ | NP | TTF1+ |
| **LCNEC1** | Large cell | Intermediate | Oval, stippled chromatin | Pinpoint to prominent | Pale granular | Expansive, sheets to organoid nests | - | +++ | +++ | +++ | TTF1+ |

| **LCNEC2** | Large cell | Low | Oval, stippled chromatin | Pinpoint to prominent | Pale granular | Infiltrative, organoid nests | + | +++ | +++ | +++ | NSE+ TTF1- |
| --- | --- | --- | --- | --- | --- | --- | --- | --- | --- | --- | --- |
| **LCNEC3** | Large cell | Intermediate | Pleomorphic, stippled chromatin | Pinpoint to prominent | Pale granular | Infiltrative, sheets and cords | - | +++ | ++ | - | NSE+ TTF1+ |
| **LCNEC4** | Large cell | Low | Round/oval, plasmacytoid | Pinpoint to prominent | Clear to eosinophilic | Circumscribed, solid, organoid, focal cords | + | +++ | - | - | NSE+ TTF1- |

*SYN: Synaptophysin, CHR: Chromogranin, NP: Not performed*

*SYN, CHR, INSM1: +++≥90% staining; ++50-89% staining; +<50% staining*

*TTF1, NSE: +positive, -negative*

**Supplementary Table S3.** Clinicopathologic features and neuroendocrine marker expression of grade 3 neuroendocrine tumors and invasive breast carcinomas with neuroendocrine differentiation.

| **Case ID** | **Age (y)** | **Diagnosis** | **Grade** | **Size (cm)** | **DCIS** | **LVI** | **LN status** | **SYN** | **CHR** | **INSM1** | **ER** | **PR** | **HER2** | **Ki67** | **Treatment** | **Follow-up (m)** | **Relevant cancer history** |
| --- | --- | --- | --- | --- | --- | --- | --- | --- | --- | --- | --- | --- | --- | --- | --- | --- | --- |
| **NET1** | 61 | NET | 3 | 5.1 (y) | - | + | + (1/9) | +++ | + | ++ | + | + | - | 20-30% | Neo chemotherapy, lumpectomy, radiation | NED (22) |  |
| **NET2** | 80 | NET | 3 | 1.6 | - | - | - (0/1) | +++ | ++ | ++ | + | + | - | 30-40% | Lumpectomy, radiation, briefly endocrine | NED (31) | Lung adenocarcinoma 8 y prior (lobectomy) |
| **NET3** | 59 | NET | 3 | 0.8 (y) | + | - | + (1/7) | +++ | + | + | + | + | - | 20-30% | Neo chemotherapy, lumpectomy, recurrence treated with neo chemotherapy, lumpectomy, endocrine | NED (28) | Recurrence 14 m following surgery |
| **NET4** | 86 | NET | 3 | 3.6 | + | + | NP | +++ | + | +++ | + | + | - | NP | Lumpectomy, endocrine | NED (22) |  |
| **NET5** | 49 | NET | 3 | 4.6 (y) | + | + | + (18/19) | +++ | ++ | +++ | + | + | - | 15-20% | Neo chemotherapy and endocrine, lumpectomy, radiation, endocrine | NED (3) |  |
| **NET6** | 53 | NET | 3 | 3.1 | + | + | - (0/7) | +++ | + | +++ | + | + | - | 15-20% | Lumpectomy, chemotherapy, radiation, endocrine | NED (88) | Lung WDNET 6 y later (surgery) |
| **NET7** | 51 | NET | 3 | 2 (y) | + | + | NP | +++ | + | NP | + | - | - | 80% | Neo endocrine, radiation to spine met, lumpectomy, radiation, endocrine | AWD (37) |  |
| **NET8** | 73 | NET | 3 | 5 | + | + | + (3/14) | +++ | +++ | NP | + | + | - | 15% | Lumpectomy, chemotherapy, radiation, endocrine | NED (25) |  |
| **IDCNED1** | 91 | IDC-NED | 3 | 0.7† | -† | -† | NP | + | - | ++ | - | - | - | 70% | LFU | LFU |  |
| **IDCNED2** | 39 | IDC-NED | 3 | 2 (i) | + | -† | NP | + | - | - | + | + | - | 20% | Liver met resection showing IDC-NST, no breast surgery, chemotherapy, endocrine | AWD (65) | Ipsilateral IDC-NST 6 y prior (lumpectomy, radiation, chemotherapy, endocrine) |
| **ILCNED** | 59 | ILC-NED | 3 | 3.6 | - (ALH) | - | + (1/1) | ++ | - | ++ | + | + | - | 15% | Mastectomy, endocrine | NED (8) |  |

*AWD: Alive with disease; CHR: Chromogranin; DCIS: Ductal carcinoma in situ; IDCNED: Invasive ductal carcinoma with neuroendocrine differentiation; ILCNED: Invasive lobular carcinoma with neuroendocrine differentiation; LFU: Lost to follow-up; LN: Lymph node; LVI: Lymphovascular invasion; NED: No evidence of disease; Neo: Neoadjuvant; NET: Neuroendocrine tumor; NP: Not performed; SYN: Synaptophysin*

*SYN, CHR, INSM1: +++≥90% staining, ++50-89% staining, +<50% staining*

*† based on core biopsy alone*

*(y) post-neoadjuvant chemotherapy*

*(i) based on imaging; never underwent surgery*

**Supplementary Table S4.** Chemotherapeutic agents used in the treatment of patients with NEC.

| **Case ID** | **Chemotherapeutic agents** |
| --- | --- |
| SCNEC1 | platinum/**etoposide** |
| SCNEC2 | carboplatin/paclitaxel, cisplatin/**etoposide** |
| SCNEC3 | cisplatin/**etoposide** |
| SCNEC4 | doxorubicin/cyclophosphamide |
| SCNEC5 | 5-FU/**etoposide**/cyclophosphamide, paclitaxel |
| SCNEC6 | doxorubicin/cyclophosphamide, cisplatin/**etoposide** |
| SCNEC7 | carboplatin/**etoposide**, carboplatin/paclitaxel |
| ANEC1 | doxorubicin/cyclophosphamide-paclitaxel |
| ANEC2 | docetaxel/cyclophosphamide, carboplatin/**etoposide** |
| LCNEC1 | carboplatin/docetaxel |
| LCNEC2 | docetaxel/carboplatin/trastuzumab/pertuzumab, doxorubicin/cyclophosphamide, trastuzumab deruxtecan |
| LCNEC3 | doxorubicin/cyclophosphamide-paclitaxel, carboplatin/paclitaxel/trastuzumab, neratinib |
| LCNEC4 | unknown |

**Supplementary Table S5.** All somatic mutations detected by UCSF500 in paired tumor/normal cases.

| **Case ID** | **CHROM** | **POS** | **REF** | **ALT** | **Variant** | **Transcript ID** | **Coverage** | **MAF** |
| --- | --- | --- | --- | --- | --- | --- | --- | --- |
| **SCNEC1** | chr17 | 7579268 | 816bp deletion of intron 4 - exon 5 | | TP53 p.Y126_R158del | NM_000546 | 535 | 80% |
|  | chr13 | 48947595 | T | TCAACCTTA | RB1 p.D394fs | NM_000321 | 284 | 74% |
|  | chr19 | 42795716 | C | G | CIC p.S902* | NM_015125 | 789 | 92% |
|  | chrX | 66766450 | C | T | AR p.Q488* | NM_000044 | 610 | 20% |
|  | chr17 | 37686952 | C | G | CDK12 p.L1286V | NM_016507 | 1837 | 13% |
|  | chr8 | 77766408 | C | A | ZFHX4 p.D2417E | NM_024721 | 1562 | 40% |
|  | chr8 | 77618016 | A | T | ZFHX4 p.S565C | NM_024721 | 1060 | 50% |
|  | chr11 | 92616556 | G | C | FAT3 p.E4312Q | NM_001008781 | 700 | 24% |
|  | chr17 | 48266368 | C | T | COL1A1 p.E981K | NM_000088 | 671 | 14% |
|  | chr12 | 12038897 | C | T | ETV6 p.A397V | NM_001987 | 669 | 69% |
|  | chr17 | 79564298 | C | G | NPLOC4 p.K322N | NM_017921 | 668 | 30% |
|  | chr6 | 42110145 | T | C | C6orf132 p.K13R | NM_001164446 | 603 | 13% |
|  | chr19 | 18277030 | G | C | PIK3R2 p.E493Q | NM_005027 | 588 | 88% |
|  | chr1 | 179076920 | C | G | ABL2 p.G1161R | NM_007314 | 565 | 87% |
|  | chrX | 47040711 | G | T | RBM10 p.G449V | NM_005676 | 507 | 20% |
|  | chr12 | 78571429 | G | T | NAV3 p.R1776L | NM_001024383 | 408 | 70% |
|  | chr10 | 60027280 | C | A | IPMK p.G31V | NM_152230 | 392 | 28% |
|  | chr20 | 36012787 | G | C | SRC p.Q77H | NM_005417 | 388 | 16% |
|  | chr6 | 99283146 | C | A | POU3F2 p.Q133K | NM_005604 | 228 | 53% |
| **SCNEC2** | chr17 | 7577568 | C | A | TP53 p.C238F | NM_000546 | 547 | 76% |
|  | chr13 | 49039133 | G | C | RB1 c.2212-1G>C | NM_000321 | 151 | 64% |
|  | chr3 | 178936094 | C | A | PIK3CA p.Q546K | NM_006218 | 950 | 65% |
|  | chr4 | 55593600 | C | G | KIT p.Q556E | NM_000222 | 462 | 65% |
|  | chr15 | 93486235 | A | G | CHD2 p.Q330R | NM_001271 | 1063 | 20% |
|  | chr9 | 139417424 | C | T | NOTCH1 p.R207H | NM_017617 | 457 | 56% |
| **SCNEC3** | chr13 | 48923157 | AAGG | A | RB1 p.K202fs | NM_000321 | 175 | 97% |
|  | chr10 | 89720798 | GTACT | G | PTEN p.V317fs | NM_000314 | 251 | 91% |
| **SCNEC4** | chr17 | 7576852 | C | T | TP53 c.993+1G>A | NM_000546 | 262 | 87% |
|  | chr16 | 50788336 | G | T | CYLD c.913+1G>T | NM_015247 | 174 | 87% |
|  | chr8 | 48739367 | G | A | PRKDC p.A2878V | NM_006904 | 186 | 44% |
|  | chr17 | 7577141 | C | T | TP53 p.G266E | NM_000546 | 82 | 86% |
|  | chr13 | 49037866 | G | C | RB1 c.2107-1G>C | NM_000321 | 81 | 84% |
| **SCNEC5** | chr16 | 68857311 | C | CT | CDH1 p.S649fs | NM_004360 | 70 | 69% |
|  | chr17 | 16004584 | AT | A | NCOR1 p.D890fs | NM_006311 | 117 | 79% |
|  | chr19 | 31767556 | C | T | TSHZ3 p.R1048Q | NM_020856 | 270 | 58% |
| **SCNEC6** | chr17 | 7578211 | C | A | TP53 p.R213L | NM_000546 | 350 | 85% |
|  | chr13 | 48923142 | C | CA | RB1 p.T197fs | NM_000321 | 109 | 78% |
|  | chr9 | 80537095 | G | T | GNAQ p.Y101* | NM_002072 | 70 | 7% |
|  | chr6 | 152738069 | G | A | SYNE1 p.R1842T | NM_033071 | 329 | 44% |
|  | chr2 | 61147587 | T | C | REL p.L331S | NM_002908 | 81 | 9% |
| **ANEC1** | chr17 | 7578532 | A | T | TP53 p.M133K | NM_000546 | 153 | 96% |
|  | chr11 | 92533189 | T | A | FAT3 p.F2337Y | NM_001008781 | 245 | 51% |
|  | chr14 | 75514897 | T | A | MLH3 p.K488L | NM_014381 | 317 | 26% |
|  | chr3 | 137742517 | C | T | CLDN18 p.R80* | NM_016369 | 85 | 5% |
|  | chr3 | 183273417 | C | T | KLHL6 p.A9T | NM_130446 | 87 | 5% |
| **ANEC2 (NEC)** | chr17 | 7577539 | G | A | TP53 p.R248W | NM_000546 | 683 | 94% |
|  | chr3 | 178916944 | A | G | PIK3CA p.K111E | NM_006218 | 704 | 47% |
|  | chr14 | 95566157 | T | C | DICER1 p.Q1389R | NM_030621 | 796 | 46% |
|  | chr15 | 42028565 | A | G | MGA p.K1368R | NM_001080541 | 506 | 47% |
| **ANEC2 (IDCNST)** | chr17 | 7577539 | G | A | TP53 p.R248W | NM_000546 | 450 | 31% |
|  | chr3 | 178916944 | A | G | PIK3CA p.K111E | NM_006218 | 592 | 24% |
|  | chr14 | 95566157 | T | C | DICER1 p.Q1389R | NM_030621 | 671 | 20% |
|  | chr15 | 42028565 | A | G | MGA p.K1368R | NM_001080541 | 448 | 19% |
| **LCNEC1 (LCNEC)** | chr17 | 7574003 | G | A | TP53 p.R342* | NM_000546 | 327 | 76% |
|  | chr13 | 48890105 | 160777bp duplication exons 3-25 | | RB1 | NM_000321 | N/A | N/A |
|  | chr12 | 4479778 | C | A | FGF23 p.E163* | NM_020638 | 1611 | 12% |
|  | chr7 | 101847752 | G | C | CUX1 p.E997Q | NM_181552 | 697 | 25% |
|  | chr14 | 81422193 | C | A | TSHR p.L57M | NM_000369 | 532 | 74% |
| **LCNEC1 (IDCNST)** | chr17 | 7574003 | G | A | TP53 p.R342* | NM_000546 | 402 | 39% |
|  | chr13 | 48890105 | 160777bp duplication exons 3-25 | | RB1 | NM_000321 | N/A | N/A |
|  | chr7 | 6426892 | C | T | RAC1 p.P29S | NM_006908 | 625 | 4% |
|  | chr1 | 201983076 | G | A | ELF3 p.E309K | NM_001114309 | 970 | 3% |
|  | chr20 | 39742725 | A | G | TOP1 p.Y523C | NM_003286 | 880 | 5% |
|  | chrX | 76849167 | C | G | ATRX p.V2037L | NM_000489 | 527 | 3% |
|  | chr7 | 101847752 | G | C | CUX1 p.E997Q | NM_181552 | 559 | 13% |
|  | chr14 | 81422193 | C | A | TSHR p.L57M | NM_000369 | 750 | 30% |
| **LCNEC3** | chr2 | 212426692 | A | G | ERBB4 p.V808A | NM_005235 | 271 | 19% |
| **LCNEC4** | chr16 | 68849648 | GGAACAGAAAATAACGTAAGTGT | G | CDH1 p.E518fs | NM_004360 | 140 | 74% |
|  | chr1 | 241682991 | G | A | FH p.S11L | NM_000143 | 342 | 34% |
|  | chr9 | 98238407 | G | T | PTCH1 p.A546D | NM_000264 | 374 | 6% |

*bp: Basepair; IDCNST: Invasive ductal carcinoma, no special type component; LCNEC: Large cell neuroendocrine carcinoma; MAF: Mutant allele frequency; NEC: Neuroendocrine carcinoma component; SCNEC: Small cell neuroendocrine carcinoma*

**Supplementary Table S6.** All alterations reported by FoundationOne for cases SCNEC7 and LCNEC2.

| **Case ID** | **Chromosome** | **Alteration** | **Variant** | **Transcript ID** | **MAF** | **Pathogenic** |
| --- | --- | --- | --- | --- | --- | --- |
| **SCNEC7** | chr10 | Loss of exons 1-6 | PTEN | NR | NA | x |
|  | chr12 | Amplification | MDM2 | NR | NA | x |
|  | chr1 | Amplification | MDM4 | NR | NA | x |
|  | chr8 | Amplification | ZNF703 | NR | NA | x |
|  | chr1 | Amplification | IKBKE | NR | NA |  |
|  | chr1 | Amplification | PIK3C2B | NR | NA |  |
|  | chr1 | Equivocal amplification | NTRK1 | NR | NA |  |
|  | chr8 | Amplification | LYN | NR | NA |  |
|  | chr8 | Amplification | NSD3 | NR | NA |  |
|  | chr8 | Amplification | RAD21 | NR | NA |  |
|  | chr8 | Equivocal amplification | FGFR1 | NR | NA |  |
| **LCNEC2** | chr17 | 488A>G | TP53 Y163C | NM_000546 | 72% | x |
|  | chr13 | 974_975delAT | RB1 p.Y325fs*2 | NM_000321 | 63% | x |
|  | chr3 | 1636C>A | PIK3CA p.Q546K | NM_006218 | 38% | x |
|  | chr13 | 712G>A | DIS3 p.D238N | NM_001128226 | 45% |  |
|  | chr13 | NR | DIS3 p.T869R | NM_001128226 | NR |  |
|  | chr17 | NR | FLCN p.A324V | NR | NR |  |
|  | chr1 | NR | HSD3B1 p.S330fs*1 | NR | NR |  |
|  | chr8 | NR | NBN p.T402A | NR | NR |  |
|  | chr9 | NR | PAX5 p.A376S | NR | NR |  |
|  | chr2 | NR | REL S588I | NR | NR |  |
|  | chr5 | NR | RICTOR p.Y490fs*20 | NR | NR |  |

*MAF: Mutant allele frequency; SCNEC: Small cell neuroendocrine carcinoma; LCNEC: Large cell neuroendocrine carcinoma; NA: Not applicable; NR: Not reported*

**Supplementary Table S7.** Mean target coverage of cases sequenced with UCSF500.

| **Case ID** | **RPA** | **CGP** | **Component** | **Mean target coverage** |
| --- | --- | --- | --- | --- |
| SCNEC1 | 869 | 3064 | SCNEC | 662 |
| SCNEC2 | 872 | 3165 | SCNEC | 673 |
| SCNEC3 | 896 | 3149 | SCNEC | 665 |
| SCNEC4 | 894 | 3147 | SCNEC | 342 |
| SCNEC5 | 870 | 3058 | SCNEC | 180 |
| SCNEC6 | 891 | 3140 | SCNEC | 247 |
| ANEC1 | 864 | 3020 | NEC | 231 |
| ANEC2 (NEC) | 873 | 3167 | NEC | 674 |
| ANEC2 (IDCNST) | 873 | 3168 | IDC-NST | 558 |
| LCNEC1 (LCNEC) | 1934 | 8654 | LCNEC | 813 |
| LCNEC1 (IDCNST) | 1934 | 8653 | IDC-NST | 767 |
| LCNEC3 | 868 | 3062 | LCNEC | 226 |
| LCNEC4 | 866 | 3051 | LCNEC | 535 |

*IDCNST: Invasive ductal carcinoma, no special type component; LCNEC: Large cell neuroendocrine carcinoma; NEC: Neuroendocrine carcinoma component; SCNEC: Small cell neuroendocrine carcinoma*

**Supplementary Table S8.** Selected immunohistochemistry and in situ hybridization of all grade 3 neuroendocrine carcinomas, neuroendocrine tumors, and invasive breast carcinomas with neuroendocrine differentiation.

| **Case ID** | **Diagnosis** | **RBⱡ** | **p53*** | **p16*** | **HPV ishⱡ** | **GATA3ⱡ** | **ATRXⱡ** |
| --- | --- | --- | --- | --- | --- | --- | --- |
| **SCNEC1** | SCNEC | - | - | ++ | - | - | + |
| **SCNEC2** | SCNEC | - | NP | NP | NP | - | + |
| **SCNEC3** | SCNEC | - | - | ++ | - | - | + |
| **SCNEC4** | SCNEC | - | - | ++ | - | - | + |
| **SCNEC5** | SCNEC | - | + | ++ | - | NP | + |
| **SCNEC6** | SCNEC | NP | NP | NP | NP | + | NP |
| **SCNEC7** | SCNEC | - | + | ++ | - | + | + |
| **ANEC1** | NEC, ambiguous | - | ++ | ++ | - | - | + |
| **ANEC2** | NEC, ambiguous | - | NP | ++ | - | - | + |
|  | IDCNST | + | NP | + | - | + | + |
| **LCNEC1** | LCNEC | - | ++ | ++ | - | - | + |
|  | IDCNST | - | ++ | ++ | - | + | + |
| **LCNEC2** | LCNEC | - | ++ | ++ | - | - | + |
| **LCNEC3** | LCNEC | + | + | + | - | + | + |
| **LCNEC4** | LCNEC | + | + | + | - | + | + |
| **NET1** | NET | + | + | + | - | + | + |
| **NET2** | NET | + | + | + | - | + | NP |
| **NET3** | NET | + | + | - | - | + | + |
| **NET4** | NET | + | + | - | - | + | + |
| **NET5** | NET | + | + | + | - | + | + |
| **NET6** | NET | + | + | + | - | + | + |
| **NET7** | NET | + | + | + | NP | NP | + |
| **NET8** | NET | + | + | NP | NP | + | + |
| **IDCNED1** | IDCNED | + | + | + | - | - | + |
| **IDCNED2** | IDCNED | + | + | + | - | + | + |
| **ILCNED** | ILCNED | + | + | + | - | + | + |

**p53 and p16: -Negative, +Positive, non-diffuse (wild-type), ++Positive, diffuse (≥90%)*

*ⱡRB, GATA3, ATRX, HPV ish: +Positive, -Negative*

*HPV ish: High-risk human papillomavirus in situ hybridization; IDCNED: Invasive ductal carcinoma with neuroendocrine differentiation; IDCNST: Invasive ductal carcinoma, no special type component; ILCNED: Invasive lobular carcinoma with neuroendocrine differentiation, solid type; LCNEC: Large cell neuroendocrine carcinoma; NEC: Neuroendocrine carcinoma component; NET: Neuroendocrine tumor (grade 3); SCNEC: Small cell neuroendocrine carcinoma*
