# Supplementary material for: Genetic and immunohistochemical profiling of small cell and large cell neuroendocrine carcinomas of the breast
Source: Mod Pathol. 2022 May 19;35(10):1349–61. doi: 10.1038/s41379-022-01090-y (PMC9514991; doi:10.1038/s41379-022-01090-y)
Supplement: Supplementary file 2 — Supplementary Figures [file 41379_2022_1090_MOESM2_ESM.pdf]

Supplementary figure legends

Supplementary Figure S1. Small cell neuroendocrine carcinoma and large cell neuroendocrine carcinoma with lobular differentiation. **A, B** SCNEC5 and **C, D** LCNEC4, with single-file growth patterns and negative immunohistochemistry for E-cadherin (**B,D**).

Supplementary Figure S2. Additional Nottingham grade 3 neuroendocrine tumors and invasive breast carcinomas with neuroendocrine differentiation. **A-C** NET4, with diffusely positive synaptophysin expression (**B**) and positive INSM1 (**C**) immunostaining. **D-F** Invasive ductal carcinoma with neuroendocrine differentiation (IDC-NED2), with patchy positive synaptophysin (**E**) and positive ER (**F**) expression. **G-I** Invasive lobular carcinoma with neuroendocrine differentiation (ILC-NED), with patchy positive INSM1 (**H**) and aberrant E-cadherin (**I**) expression.

Supplementary Figure S3. *RB1* and *TP53* co-alteration in neuroendocrine carcinomas compared to Nottingham grade 3 invasive ductal carcinomas of no special type. *$p<0.05$

Supplementary Figure S4. Mixed large cell neuroendocrine carcinoma and invasive ductal carcinoma of no special type. **A** LCNEC1, comprised of mixed components of LCNEC and IDC-NST (*left*, IDC-NST; *right*, LCNEC). **B** Synaptophysin highlights the NEC component. **C,D** Higher power images of IDC-NST (**C**) and LCNEC (**D**). **E** Chromosomal copy number plots reveal multiple gains and losses that are shared between the IDC-NST and LCNEC components, with additional alterations only in LCNEC (*red arrows*).

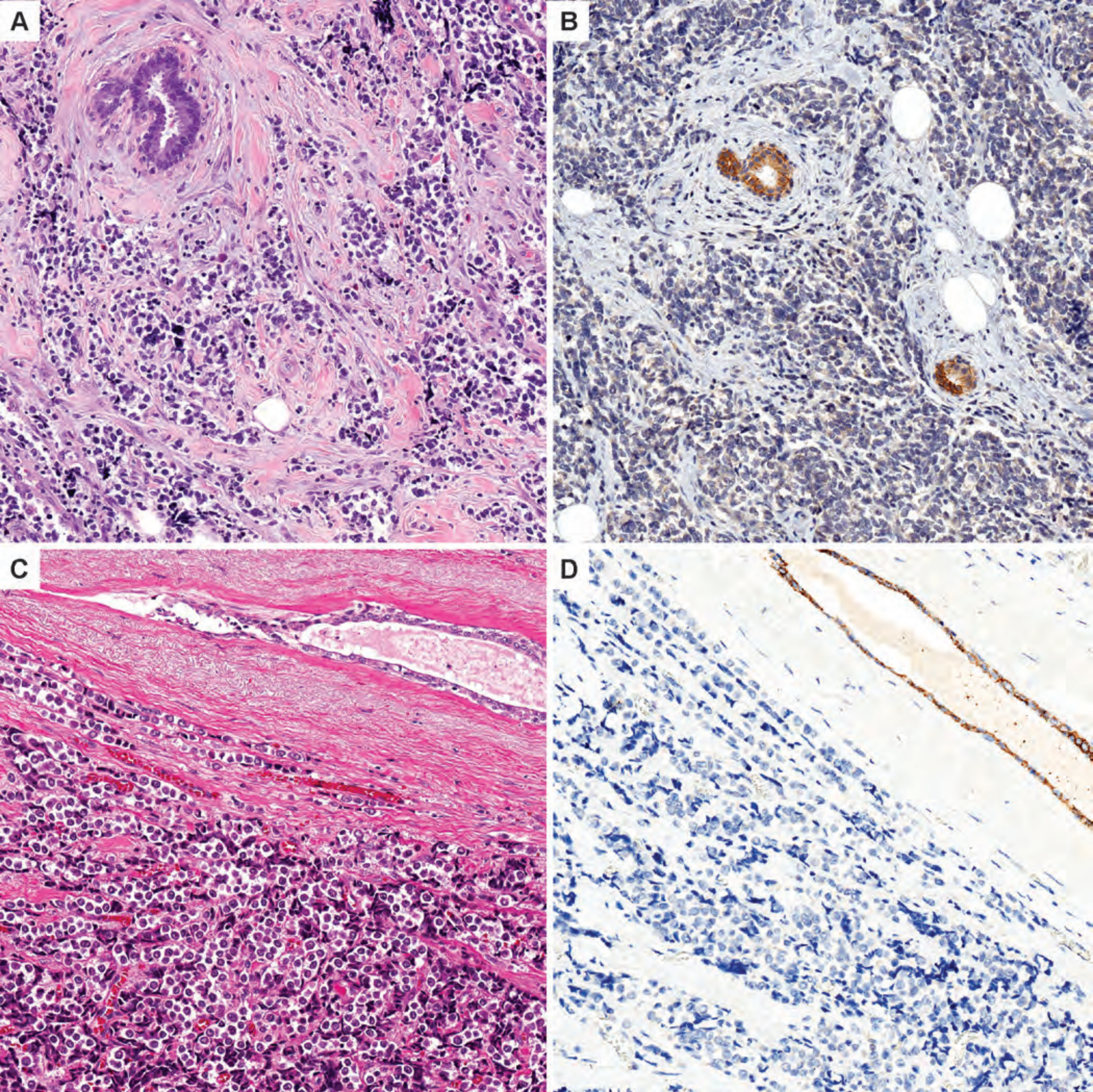
A
B
C
D

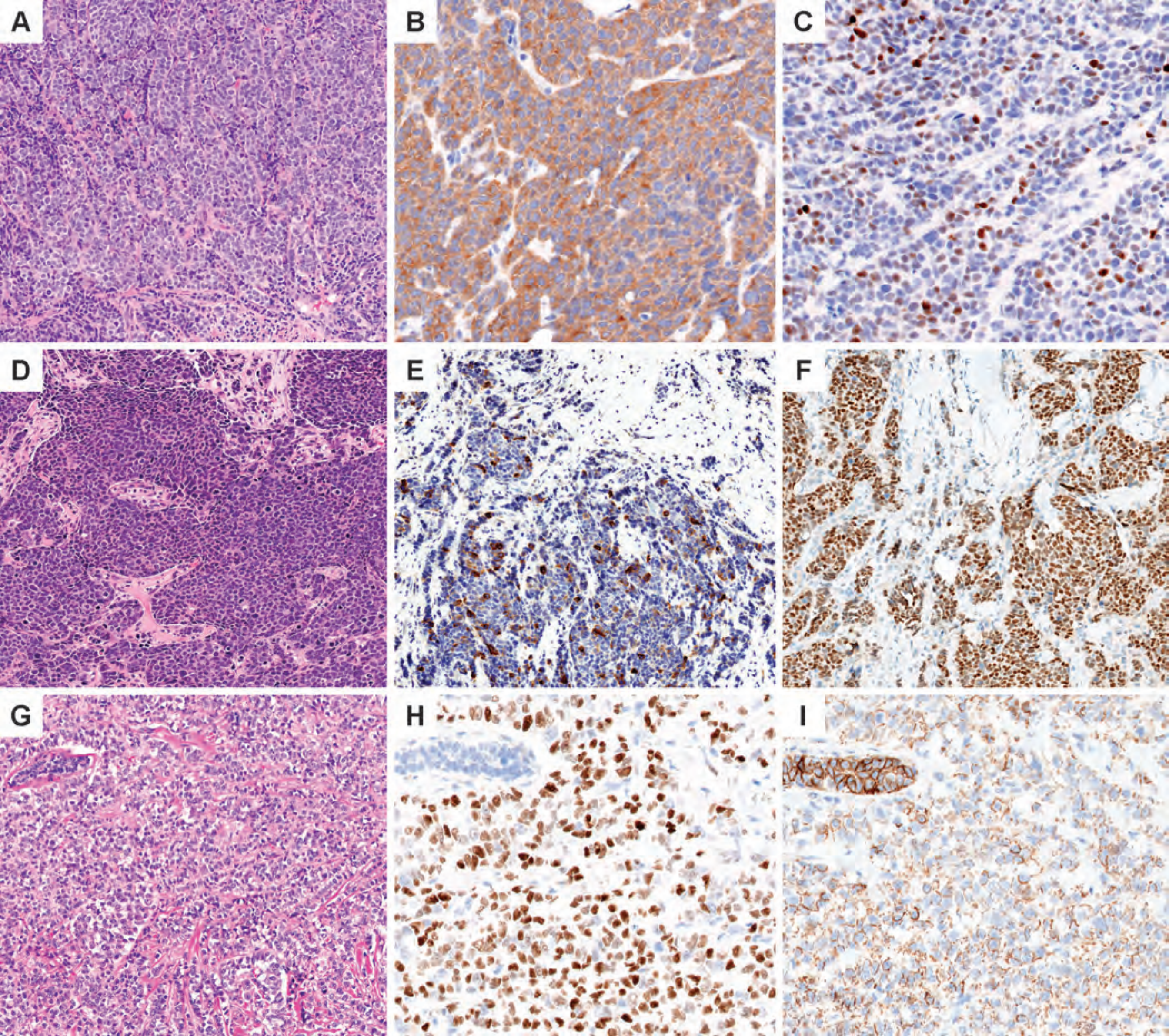
A
B
C
D
E
F
G
H
I

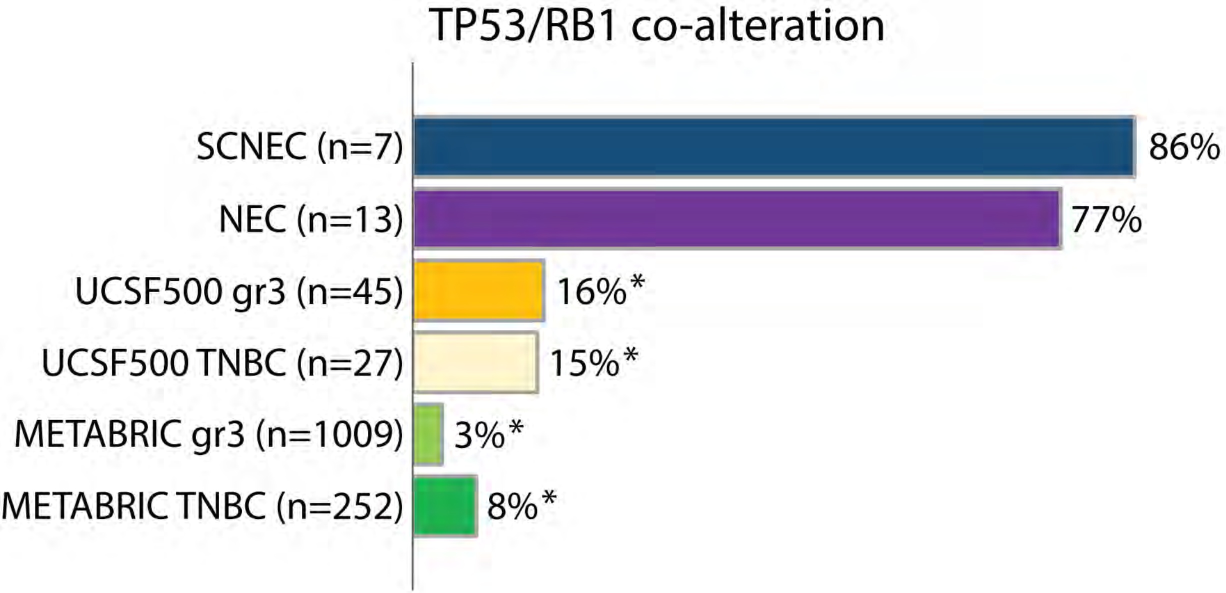
TP53/RB1 co-alteration
SCNEC (n=7)
86%
NEC (n=13)
77%
UCSF500 gr3 (n=45)
16%*
UCSF500 TNBC (n=27)
15%*
METABRIC gr3 (n=1009)
3%*
METABRIC TNBC (n=252)
8%*

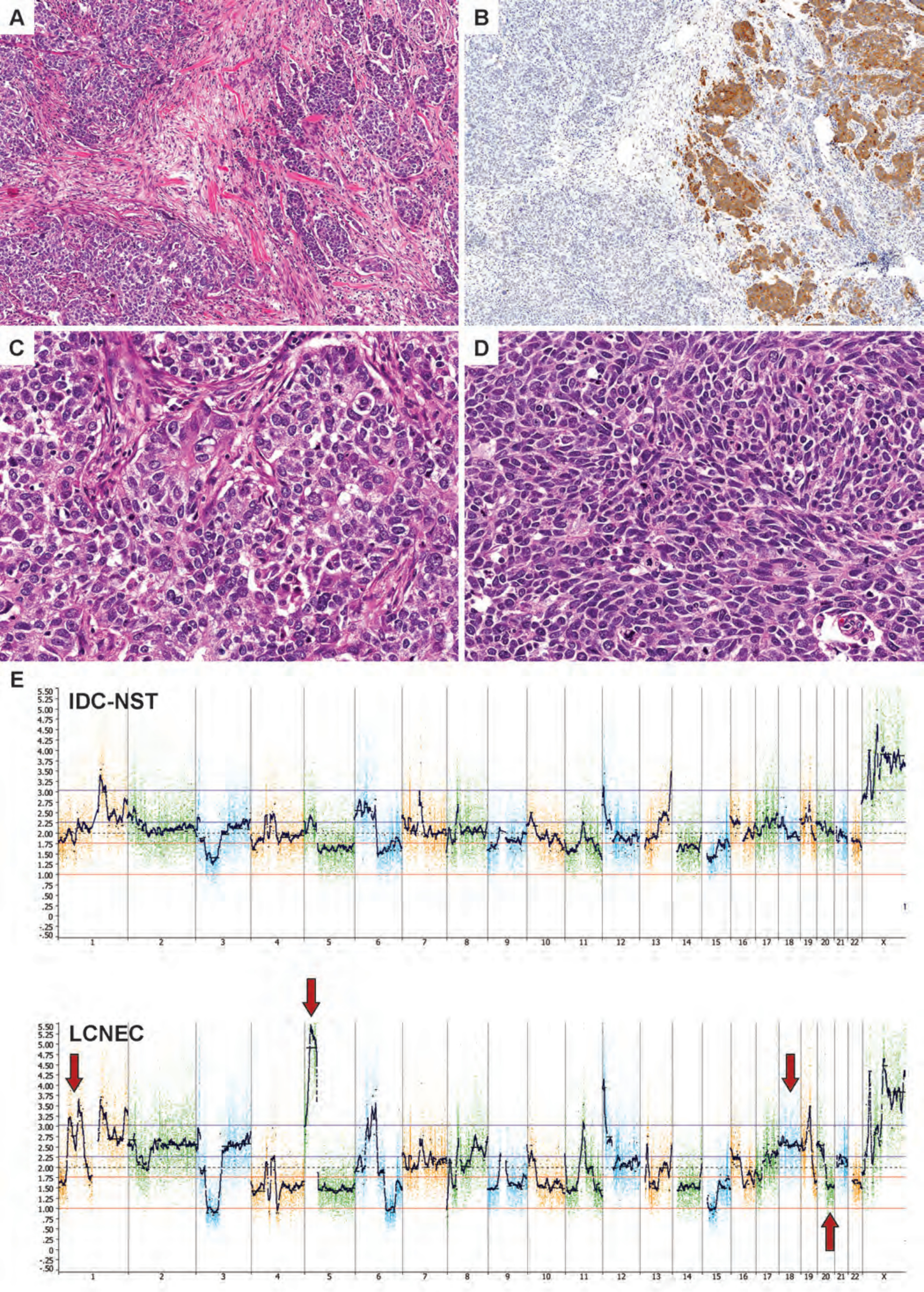

A
B
C
D
E
IDC-NST
LCNEC
5.50 5.25 5.00 4.75 4.50 4.25 4.00 3.75 3.50 3.25 3.00 2.75 2.50 2.25 2.00 1.75 1.50 1.25 1.00 .75 .50 .25 0 -.25 -.50
1 2 3 4 5 6 7 8 9 10 11 12 13 14 15 16 17 18 19 20 21 22 X
